# Professional duties are now considered legal duties of care within genomic medicine

**DOI:** 10.1038/s41431-020-0663-3

**Published:** 2020-06-08

**Authors:** Middleton Anna, Patch Christine, Roberts Jonathan, Milne Richard, Costa Alessia, Robarts Lauren, Atutornu Jerome

**Affiliations:** 1 Society and Ethics Research, Connecting Science, Wellcome Genome Campus, Cambridge, UK; 2 Faculty of Education, University of Cambridge, Cambridge, UK; 3 Genomics England, London, UK; 4 East of England Genomic Medicine Service, Cambridge, UK; 5 Institute of Public Health, University of Cambridge, Cambridge, UK; 6 School of Health and Sports Science, University of Suffolk, Ipswich, UK

**Keywords:** Medical ethics, Social sciences

## Abstract

The legal duty to protect patient confidentiality is common knowledge amongst healthcare professionals. However, what may not be widely known, is that this duty is not always absolute. In the United Kingdom, both the General Medical Council governing the practice of all doctors, as well as many other professional codes of practice recognise that, under certain circumstances, it may be appropriate to break confidentiality. This arises when there is a wider duty to protect the health of others, and when the risk of non-disclosure outweighs the potential harm from breaking confidentiality. We discuss this situation specifically in relation to genomic medicine where relatives in a family may have differing views on the sharing of familial genetic information. Overruling a patient’s wishes is predicated on balancing the duty of care towards the patient versus protecting their relative from serious harm. We discuss the practice implications of a pivotal legal case that concluded recently in the High Court of Justice in England and Wales, *ABC* v *St Georges Healthcare NHS Trust & Ors*. Professional guidance is already clear that genetic healthcare professionals must undertake a balancing exercise to weigh up contradictory duties of care. However, the judge has provided a new legal weighting to these professional duties: ‘The scope of the duty extends not only to conducting the necessary balancing exercise but also to acting in accordance with its outcome’ [1: 189]. In the context of genomic medicine, this has important consequences for clinical practice.

## Overview

The pivotal *ABC* v *St Georges Healthcare NHS Trust and Ors*^1^ case has reached its conclusion in the High Court of Justice in England and Wales [1]. The case has been debated extensively [2–4] and involves ABC (who is a daughter of a man with Huntington’s disease, HD). The man killed his wife and whilst in the care of psychiatric prison services received his diagnosis of HD. He expressly forbade his doctors from telling ABC about this inherited condition because she was pregnant at the time and he was worried she would have a termination of pregnancy if she knew. ABC sued the NHS trust caring for her father, claiming that her father’s clinical team had a duty of care to share his diagnosis with her even though he explicitly did not consent to this. ABC said that, had she known, she would have indeed had a termination of her pregnancy. The judge, Mrs Justice Yip in this case was unconvinced by her argument, and for this reason plus others, ABC did not win her case. However, despite this, the judge’s evaluation of ABC’s situation has clear implications for current clinical practice, specifically within Clinical Genetics and Genetic Counselling Services. As here, genetic healthcare professionals often have a close clinical proximity to multiple members of the same family.

Mrs Justice Yip concluded that it is ‘fair, just and reasonable’ [1:188] to impose a legal duty to conduct a balancing act between the interests of the claimant and that of the father’s confidentiality, and importantly that ‘The scope of the duty extends not only to conducting the necessary balancing exercise but also to acting in accordance with its outcome.’ [1:189].

Thus, if the need to warn relatives of their risk of disease appears to outweigh wishes of the primary patient (‘proband’) not to support this, then as a result of Mrs Justice Yip’s conclusions, healthcare professionals now have a legal duty to act on this and warn relatives.

The latest 2019 Joint Committee of Genomics in Medicine Professional Recommendations [5] (earlier versions of which were quoted in the court proceedings) on Consent and Confidentiality in genomic medicine state:

‘If breach of confidence is necessary:

i. Attempt to obtain consent to disclosure from the patient.
ii. If it is not possible to obtain consent from the patient:
  - Discuss the case with experienced professional colleagues (eg hospital clinical ethics committee, Genethics forum).
  - Tell the patient that you intend to breach this confidence and why.
  - Contact a relative where it is practical and reasonable to do so.
  - Keep any disclosure to the minimum that is strictly necessary for the communication of risk.
  - Record the balancing act undertaken and justification for breaching confidence.’ p 29

### The Unit of Care—the primary patient or their relatives

The judgement further concludes that, while courts have recognised that doctors may owe a duty of care to persons other than their primary patient, this duty can only arise ‘where there is a close proximal relationship between the claimant and defendant’ [1:170].

Within Western medicine generally, clinical services usually occur within the context of a one-on-one relationship, i.e. a health service interacts with one patient at a time, and delivery of this is facilitated through a doctor/healthcare professional-patient relationship. A patient has one set of medical records, they can expect that conversations with their healthcare providers are confidential, and, unless there are concerns about competency, a patient (with advice from their healthcare professionals) makes their own decisions about their own healthcare. Thus the ‘unit of care’ across most of medicine outside of specialist genetics services, is the primary patient.

However, within established Clinical Genetics and Genetic Counselling services, this model has evolved. It is recognised that the primary patient/proband shares genetic and health information with biological relatives, and thus the ‘unit of care’ is often seen as the family. Within the clinical genetics and bioethics literature, this is discussed in terms of a ‘joint account’—with all relatives considered as having an equal role in being able to contribute and have access to information, as well as to services [6].

Seeing the unit of care within Clinical Genetics and Genetic Counselling as the ‘family’ as opposed to solely the primary patient (‘proband’) has been common practice for more than 50 years. In the professional duties laid out by the Association of Genetic Nurses and the Counsellors in the UK, they explain: ‘Genetic Counsellors are highly skilled healthcare professionals with training and expertise in genomic medicine and counselling skills. Their role is to interpret and explain genomic information to patients and support such patients *and their families* to make use of this information’ [emphasis added, 7]. While professional duties to relatives have emerged naturally within this discipline, legal duties have not been clearly defined. The judgement in the *ABC* v *St George’s NHS Trust & Ors* case articulates that if a healthcare professional in England or Wales has a ‘close proximity’ to relatives of a primary patient, then this means that the healthcare professional now has a legal basis to consider what duties of care they should offer these relatives.

### Duties to relatives

Within Clinical Genetics and Genetic Counselling services in the UK, if a proband comes to a genetics clinic together with their biological siblings and those siblings participate in the consultation, then they would usually *become* (formally or informally) patients of the Clinical Genetics service by virtue of being in the consultation. As genetic information is shared between biological relatives, the information provided to the primary patient may be directly relevant to the other relatives; if they have participated in the consultation, this information will be discussed within the context of their own health at the same time. After the genetics appointment, the relatives would not necessarily be given their own set of medical records immediately but would certainly appear in the proband’s notes. They may also be offered their own separate clinical appointment in the future, where their own set of medical records may be made, or they may join a ‘family’ set of medical records, that will usually be kept separate from the main hospital database of patient records that includes all hospital patients (done so as to protect the confidentiality of multiple relatives). Even if siblings live at the opposite end of the country, the healthcare professional should provide information to the relatives so that they can seek out their own referral to see a local clinician.

Supporting ‘patients and families’ is a core part of what Clinical Genetics and Genetic Counselling Services offer, i.e. it is within scope of their professional practice, and given the ABC judgement, it is reasonable to suggest that the court (as they were in the ABC case) would be supportive of these professional duties having legal credibility.

### The legal duty to disclose inherited risks of disease to relatives

The *ABC* v *St George’s NHS Trust & Ors* case clearly articulates that the possibility that non-disclosure of genetic information would cause ABC significant psychological harm could have been (and indeed was) foreseen. It could be considered that ‘at-risk’ family members who may be affected by the disclosure or non-disclosure of genetic information (if their details are known to the clinical care teams) may have a legitimate interest in the familial information. This gives rise to an obligation to consider the family members’ interests.

However, Mrs Justice Yip clarifies: ‘The duty I have found is *not a free-standing duty of disclosure nor is it a broad duty of care owed to all relatives* in respect of genetic information. The legal duty recognises and runs parallel to an established professional duty and is to be exercised following the guidance of the GMC and other specialist medical bodies.’ [1:261] (emphasis added). This means that healthcare professionals are not expected to go tracking down relatives unknown to them or for whom there is no ‘proximity’. Particularly within routine genetic counselling services, where multiple relatives are often seen together, a ‘proximity of care’ is obvious.

When assessing the implications of this ruling, further clarity, however, is needed regarding the specifics of proximity. A Clinical Genetics team can ‘know’ a patient on different levels. In other words there are varying degrees of professional and physical proximity. For example:

- An individual genetic healthcare professional seeing multiple members of the same family in a consultation. Close proximity is likely to be established here.
- An individual genetic healthcare professional who is told by the proband the personal details of their relatives (names, dates of birth and addresses etc). Close proximity is unclear in this situation as whilst the genetic healthcare professional has never met these relatives, they have been given enough information to be able to contact them (there is no need to ‘track them down’ and it would be easy enough to make contact). Given that within the professional code of practice, genetic counsellors care for both ‘patients and their families’ [7], it would be expected that as part of their duty to such relatives, they would at least make an attempt to try to contact them. In reality professional practice varies in relation to this. It would be usual for genetic healthcare professionals to encourage the proband to pass on information to their relatives. But if they refused to do this, and if the genetic healthcare professional deemed on the basis of the balancing exercise that the relative should be told, then (as per the JCGM professional recommendations) they should contact them (as long as it is reasonable and practical to do) without the need to establish a different sort of proximity.
- A regional clinical genetics service may see multiple members of the same family. In this case they may be ‘known’ to several different clinicians within the team. Close proximity is established between the service and the family but not necessarily between individual clinicians and specific relatives. Here, it would be helpful to clarify if ‘proximity’ can only be established, one-to-one, between one clinician and one patient, or whether ‘proximity’ could apply to service delivery. Further, what happens if that service delivery is across many geographical areas? Here, Mrs Justice Yip states that there cannot be a duty of care to a patient that the clinician has never met; however, there are situations in which this seems difficult to maintain. For example a doctor would act on a duty of care to the passengers of an airline if he knew that the pilot had a risk of crashing the plane (and indeed in this case the doctor has no direct proximity to any of the passengers).

Defining a duty to perform a balancing act on grounds of proximity is a reasonable approach. However, given the shared (familial) nature of genetic information and the idiosyncratic delivery of genetics services, driven by the ‘family’ as the unit of care, further clarity is needed on this.

### Immediate practice implications

While highlighting the complexity of the case and the room for reasonable difference in professionals’ judgement about whether or not to disclose information, the court case set a precedent for the requirement of healthcare professionals to consider the potential duties of care to relatives. An important outcome of this case is a loud and clear message that all healthcare professionals need to do a better job of providing evidence that they have at least considered the duties of care they owe to relatives. And if the outcome of the ‘balancing act’ is that duties are identified to relatives, there is also a legal duty to address these.

This leads to direct implications for practice both when disclosure is accepted by the proband and more particularly when a clinician may be considering breaking the confidentiality of their primary patient. The decision to do so is dependent on balancing the conflicting interest of the parties.

In doing so, healthcare professionals seeing genetics patients and families must:

- Clearly document in all of the patient’s and relative’s medical records what the ‘balancing act’ has considered and who has been involved in any discussions. It is advisable to involve other professionals in the discussion, whether via a multidisciplinary team or a local clinical ethics committee.
- Clearly articulate and document the arguments for or against disclosure, together with an assessment of foreseeable psychological harm from both courses of action (i.e. disclosure versus non-disclosure, with a recognition that both positions may have psychological consequences). Mrs Justice Yip comments specifically on the psychological harm caused to ABC by not disclosing her father’s HD diagnosis to her.
- Deliver on the conclusion drawn from the balancing exercise (i.e. if the balancing exercise concludes there is a duty to warn relatives, the clinician is legally bound to do this).
- Consider how information should be disclosed to at-risk relatives and make arrangements for them to receive genetic counselling or other support to minimise the potential harm from receiving such information. Mrs Justice Yip was very critical of the doctor who inadvertently told ABC of her father’s diagnosis, recognising that disclosing the risk of inherited disease to relatives should be done sensitively and with follow up.

This cements what has been recommended in existing professional guidelines [5, 8]. The duty to protect confidentiality and the duty to warn are both recognised duties within the management of genetic data—one does not automatically trump the other; although, protecting confidentiality should always be the default in the absence of other competing duties. What is different is that there is now a legal credibility given to the professional guidance. As long as clinicians can show that they have weighed up the pros and cons of breaching confidentiality versus forewarning at-risk relatives of possible disease, and a duty to warn has been established, then legally this should be acted upon. This is a shift from the prior misunderstanding that a duty to protect confidentiality automatically trumps everything. In exceptional circumstances, there is now legal support to prioritise a different duty. In the context of genomic medicine, this has important consequences for clinical practice.

## Supplementary information

ABC v St Georges Judgement

## Footnotes

1 The full name of the case is ABC v St George’s Healthcare NHS Trust, South West London and St George’s Mental Health NHS Trust and Sussex Partnership NHS Foundation Trust. Hearing dates: 18, 20, 21, 22, 25 November 2019 & 15 January 2020. For brevity we refer to this case in summary form as ‘ABC v St George’s NHS Trust’
