# Supplementary material for: Professional duties are now considered legal duties of care within genomic medicine
Source: Eur J Hum Genet. 2020 Jun 8;28(10):1301–4. doi: 10.1038/s41431-020-0663-3 (PMC7609317; doi:10.1038/s41431-020-0663-3)
Supplement: Supplementary file 1 — ABC v St Georges Judgement [file 41431_2020_663_MOESM1_ESM.doc]

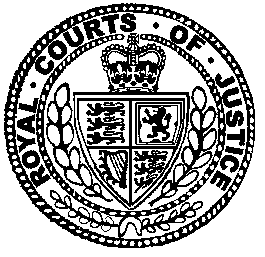


Neutral Citation Number: [2020] EWHC 455 (QB)

Case No: QB-2013-009529

IN THE HIGH COURT OF JUSTICE

**QUEEN'S BENCH DIVISION**

Royal Courts of Justice

Strand, London, WC2A 2LL

Date: 28/02/2020

**Handed down in Preston Combined Court Centre**

**Before** :

MRS JUSTICE YIP DBE

- - - - - - - - - - - - - - - - - - - - -

**Between :**

|  | **ABC** | Claimant |
| --- | --- | --- |
|  | **- and –** |  |
|  | **(1) ST GEORGE’S HEALTHCARE NHS TRUST**  **(2) SOUTH WEST LONDON AND ST GEORGE’S MENTAL HEALTH NHS TRUST**  **(3) SUSSEX PARTNERSHIP NHS FOUNDATION TRUST** | Defendant |
|  |  |  |

- - - - - - - - - - - - - - - - - - - - -

- - - - - - - - - - - - - - - - - - - - -

**Ms E.A Gumbel QC** (instructed by **Fieldfisher**) for the **Claimant**

**Mr P Havers QC & Ms H Noyce** (instructed by **Capsticks LLP**) for the **Defendant**

Hearing dates: 18, 20, 21, 22, 25 November 2019 & 15 January 2020

- - - - - - - - - - - - - - - - - - - - -

Approved Judgment

I direct that pursuant to CPR PD 39A para 6.1 no official shorthand note shall be taken of this Judgment and that copies of this version as handed down may be treated as authentic.

.............................

**Mrs Justice Yip :**

1. By this claim brought against three NHS trusts, the claimant contends that the defendants breached a duty of care owed to her and/or acted contrary to her rights under Article 8 of the European Convention on Human Rights in failing to alert her to the risk that she had inherited the gene for Huntington’s disease in time for her to terminate her pregnancy. The claimant seeks damages for the continuation of her pregnancy, psychiatric damage and consequential losses. The parties have narrowed the issues for the court’s determination by agreeing that if the court finds an actionable breach of duty on the part of the defendants (or any of them) and that, but for that breach, the claimant would have terminated her pregnancy, the claimant should recover damages in the sum of £345,000.
2. The genetic risk had been revealed to the defendants through diagnosis of the claimant’s father. He declined to consent to disclosure of the information to the claimant and the defendants’ clinicians took the view that they should not override his confidentiality. The defendants deny that, as a matter of law, they owed any relevant duty of care to the claimant. Even if such a duty was owed, they contend, on the facts of the case, that they did not breach that duty. Further, they maintain that even if there was a breach of duty, it did not cause the claimant any injury because the evidence does not establish that she would have had a termination but for the breach. The defendants say that the claim under the Human Rights Act 1998 fails for the same reasons as the common law claim.

Procedural history

1. At an early stage in the proceedings, the defendants applied to strike out the claim on the ground that it disclosed no reasonable cause of action. Nicol J acceded to that application (*ABC v St George’s Healthcare and others* [2015] EWHC 1394 (QB)). The Court of Appeal overturned his decision and ordered that the matter should proceed to trial (*ABC v St George’s Healthcare and others* [2017] EWCA Civ 336.)

Anonymity

1. As is apparent, this is an acutely sensitive case. Nicol J made an anonymity order when the matter came before him. I entirely agree with his reasons for doing so, expressed at paragraph 44 of his judgment. I would add that the Article 8 rights of the claimant’s sister, are also engaged.
2. Nicol J’s order included provision for an application by the press to set aside or vary the restrictions he imposed. No such application was made, and the anonymity order was continued through the Court of Appeal proceedings. I continued the anonymity order at the outset of the trial, no representations having been made to the contrary. I consider that the balance remains in favour of protecting the privacy of the claimant, her daughter and other immediate family members. It follows that the claimant continues to be referred to only as ABC for the purpose of this claim. The claimant’s father is referred to as XX. Consistent with the principles of open justice, the identities of the trusts and the professionals involved may be reported.

Factual background

1. The facts giving rise to the claim are both tragic and unusual. In 2007, the claimant’s father killed her mother. He was convicted of manslaughter by reason of diminished responsibility and made subject of a restricted Hospital Order pursuant to section 37 and section 41 of the Mental Health Act 1983. XX was detained at the Shaftsbury Clinic, Springfield Hospital (“Springfield”) which is run by the second defendant. He received care and treatment through a multi-disciplinary team, including psychiatrists, psychologists, other therapists and social workers. XX’s responsible clinician was initially Professor Eastman. In 2009, he was transferred into the care of the team headed by Dr Olumoroti, a consultant forensic psychiatrist.
2. From early in his admission, suspicions were raised that there might be an unspecified organic explanation for some of XX’s symptoms. He underwent MRI scans in 2007 and 2008 but no progressive changes were seen in the brain. On 28 April 2009, XX was referred by the psychiatric team to the neurological department at St George’s Hospital (which falls within the first defendant’s responsibility). The referral letter stated:

“Our main concern at the moment is that [XX] has gait difficulties. He has a non-progressive choreiform limb movement and an abnormal wide based gait with motor restlessness on sitting still.”

1. XX was seen by Dr Marion, consultant neurologist, on 24 June 2009. She found a clinical picture of hereditary choreic syndrome, which she said was likely to be Huntington’s chorea. She planned to refer XX to Dr McEntagart in the genetic department as St George’s. XX apparently agreed to genetic testing at the time. However, it is clear from the medical records that he later became hostile to any suggestion that he had Huntingdon’s disease and refused to undergo genetic testing. His intransigence about this appears from various entries in his medical records in July and August 2009. XX made it clear that he did not want the claimant and her sister to know that he was thought to have Huntington’s disease. He was aware that such knowledge could impact on their decision about whether to have children or not. Both daughters were in their 30s; neither had started a family.
2. Huntington’s disease (“HD”), also known as Huntington’s chorea (“HC”) is a neurodegenerative disorder of genetic origin. Although I shall refer to “the Huntington’s gene” as a shorthand, strictly it arises from a genetic mutation. It is an autosomal dominant trait so that the child of someone with the condition has a 50% chance of being affected. The clinical features of the condition include abnormalities of movement, cognitive problems and psychiatric symptoms. It is an incurable disease and sufferers have a reduced life expectancy. Symptoms do not appear until adulthood, typically between the ages of 30 and 50 years.
3. The first defendant’s clinical team recognised that the likelihood that XX had Huntington’s disease had significant implications for his daughters, who were known to them. Despite struggling to come to terms with their own grief and the devastation of their family, the claimant and her sister continued to support their father. The claimant attended case conferences and planning meetings about XX. Family therapy was proposed, and the claimant commenced attending sessions in March 2009. The claimant was aware that XX had been referred for a neurological opinion but, given XX’s stance, the outcome of the appointment was not shared with her.
4. By August 2009, the second defendant’s clinical team had recognised that the situation was very difficult. Their discussions also involved Mary Davies, a social worker in the community team, for whom the third defendant is responsible. On 20 August, Dr Roberts (SHO to Dr Olumoroti) wrote to Dr McEntagart seeking her advice on managing the situation. I shall return to consider that letter and Dr McEntagart’s response.
5. By unhappy coincidence, the claimant became pregnant in July 2009, that is around the time that it emerged that XX probably had Huntington’s disease. She was not in a settled relationship and was initially ambivalent about the pregnancy continuing.
6. A multidisciplinary team meeting took place on 2 September 2009. The claimant attended. Unusually, she was asked to remain outside, and it became apparent that the meeting was proceeding without her. In advance of the meeting, the claimant had told a social worker that she was pregnant but had not decided what to do. When invited into the room, the claimant was asked by Dr Olumoroti whether she had children. She was taken aback and did not share the news of her pregnancy. However, after the meeting, she did tell her father. He shared the news with his psychologist, Ms Gill, at a session the next day. He remained adamant that he did not want to tell his daughters about the Huntington’s disease diagnosis and “so jeopardise the pregnancy.” Ms Gill reported the development to the clinical team.
7. Having discovered that the claimant was pregnant and following further discussions within the clinical team, Dr Roberts wrote again to Dr McEntagart on 18 September 2009, sending the letter by fax. She acknowledged that a diagnosis of Huntington’s disease would impact on “his daughter’s decision about whether to continue with her pregnancy” and sought urgent advice in view of the pregnancy. Having received that letter, Dr McEntegart discussed XX’s case with Dr Olumoroti by telephone and followed up with a letter dated 25 September 2009.
8. I shall return to a detailed consideration of the contents of those letters and what happened thereafter. In short, XX continued to insist that his daughters were not told, suggesting that the doctors would be acting illegally if they did so. XX had shared the information about Huntington’s disease with his brother and, in October 2009, the brother attended Springfield for a discussion of the options. After that meeting, XX agreed to be seen by the genetic team. He was seen by Professor Patton at Springfield on 27 October 2009. He gave his consent for genetic testing, having specifically excluded the results going to any member of his family.
9. The results of XX’s genetic testing were reported on 9 November 2009. They confirmed that XX had Huntington’s disease. Professor Patton and a genetic counsellor attended Springfield on 10 December 2009. They informed XX of the results in the presence of Dr Olumoroti. Following the disclosure of the results, Dr Olumoroti called an emergency team meeting to discuss how XX would be supported. It was noted that he might become distressed, depressed or even suicidal. XX continued to maintain that his daughters should not be informed of the diagnosis.
10. I note that by the time XX received the confirmed diagnosis, the claimant was more than 24 weeks pregnant. It is agreed that the last date on which she could have undergone a termination was 6 December 2009.
11. The claimant had her baby in April 2010. There is no doubt that this was a happy event for her and that she dearly loves her child.
12. On 4 August 2010, a Mental Health Tribunal directed XX’s conditional discharge subject to meeting certain requirements. The claimant was concerned about this. In that context, Dr Olumoroti and a social worker visited her at home on 23 August 2010. It is accepted that Dr Olumoroti breached XX’s confidentiality and informed the claimant of XX’s diagnosis, although the precise circumstances in which that occurred are in dispute.
13. The claimant’s sister was then in the early stages of her first pregnancy. The claimant informed the clinical team of her sister’s pregnancy. The claimant decided that she did not want XX’s diagnosis to be disclosed to her sister during her pregnancy. XX also maintained that the sister should not be told. On 7 October 2010, a meeting of the St George’s Clinical Ethics Committee took place, chaired by Professor Eastman, to address the question of whether the diagnosis should be disclosed to the claimant’s sister. After discussion of the issues, a vote was taken. The minutes record that the majority supported XX’s right to confidentiality and would not disclose the information. The claimant’s sister did not therefore learn of XX’s diagnosis until after her baby was born. She has since been tested and has been found not to have the genetic mutation.
14. The claimant did not undergo genetic testing herself until 2013. Sadly, she tested positive. Medical evidence confirms that she is likely to develop symptoms within five to ten years. She is greatly distressed by that prospect and is deeply concerned for her child’s future, particularly as she is a single parent. She has been engaged in contested family proceedings in which her medical prognosis has featured. That is a further source of distress to her. Given the agreement the parties have reached as to quantification of the damages, it is unnecessary to comment on the nature and aetiology of the claimant’s psychiatric symptoms in any detail. It is worth noting though that the claimant places emphasis on her constant worry that her child may have inherited the Huntington’s gene and her concern for her child when the claimant develops symptoms. I stress that it is not the case that the claimant did not want her child. Rather, she feels that it was unfair for her to bring a child into the world in the tragic circumstances I have set out. She complains that she was deprived of the opportunity to make that choice.

The legal basis of the claim and the issues arising

1. The claim is brought both in negligence and under the Human Rights Act 1998 for breach of Article 8 of the European Convention on Human Rights. Giving judgment in the Court of Appeal in relation to the strike-out application, Irwin LJ said [65]:

“I am unconvinced that the Convention adds anything to the common law or can provide a basis for action if the common law does not do so.”

The claimant was not precluded from maintaining her Article 8 claim at trial and she did so. However, this aspect of the case was not developed in any great detail. In her closing submissions for the claimant, Ms Gumbel QC confirmed that the claim under the Human Rights Act ran parallel to the common law claim. In addition to contending that it founded a freestanding claim, Ms Gumbel argued that the Article 8 obligation was relevant to the issue of whether a duty of care existed.

1. For practical purposes and given the nature of the agreement as to damages, the claim, however framed, requires the claimant to prove that:
   1. the defendants ought to have given her sufficient information to put her on notice of the risk that she had inherited the Huntington’s gene at a time when it was open to her to terminate her pregnancy;
   2. properly advised of that risk, she would have undergone genetic testing and discovered that she had the Huntington’s gene in time to terminate her pregnancy; and
   3. she would then have had a termination.

Ms Gumbel confirmed that the court was not asked to consider any alternative basis for an award of damages if the claimant did not establish those essential elements.

1. The issues that arise then are a mix of legal and factual issues. The essential issues I must determine may be summarised as follows:
   1. Did the defendants (or any of them) owe a relevant duty of care to the claimant?
   2. If so, what was the nature and scope of that duty?
   3. Did any duty that existed, require that the claimant be given sufficient information for her to be aware of the genetic risk at a stage that would have allowed for her to undergo genetic testing and termination of her pregnancy?
   4. If a duty of care was owed, did the defendants (or any of them) breach that duty by failing to give her information about the risk that she might have a genetic condition while it was open to her to opt to terminate her pregnancy?
   5. If there was a breach of duty, did it cause the continuation of the claimant’s pregnancy when it would otherwise have been terminated? (This involves consideration of whether the claimant would in fact have had the opportunity to undergo genetic testing and a termination in time but for the breach, and whether she would have chosen to do so.)

The claimant’s alternative cases as to the existence of a duty of care

1. As initially pleaded, the claimant’s case as to the existence of a duty of care was:

“At all relevant times the First, Second and Third Defendants knew that the Claimant was the daughter of XX and that a diagnosis of Huntington’s Disease would have a direct effect on the health, welfare and life of the claimant. They each owed to the Claimant a duty of care in respect of any medical information that was relevant to her own welfare.”

That appeared to encapsulate a broad duty arising through the defendants’ possession of information relevant to the claimant’s welfare and the knowledge that the information would impact upon her.

1. Before Nicol J, the claimant sought to amend her Particulars of Claim and placed reliance on her attendance at the family therapy sessions arranged by the second defendant. Ms Gumbel developed the argument that through undergoing family therapy the claimant was, like XX, a patient of the second defendant. On that basis, it was argued that a duty of care arose through the doctor/patient relationship.
2. During the proceedings before the Court of Appeal, the claimant was permitted to further amend her Particulars of Claim, making it clear that she specifically relied upon the existence of a duty to her as a patient and/or the assumption by the defendants of responsibility for the claimant’s welfare and well-being.
3. I note that the interlocutory judgment of the Court of Appeal focused upon whether it was fair, just and reasonable to impose a duty of care towards the claimant on the defendants. For the purposes of the strike-out application only, the defendants had accepted that proximity and reasonable foreseeability could be established. The Court of Appeal hearing occurred before four recent decisions of the Supreme Court in which the existence and scope of a common law duty of care was considered (*Robinson v Chief Constable of West Yorkshire Police* [2018] UKSC 4; *James-Bowen & others v Commissioner of Police of the Metropolis* [2018] UKSC 40; *Darnley v Croydon Health Services NHS Trust* [2018] UKSC 50; *Poole Borough Council v GN & another* [2019] UKSC 25.)
4. Through those cases, the Supreme Court has stressed that there is no general principle capable of providing a practical test applicable in every situation in order to determine whether a duty of care exists and, if so, its scope. *Caparo Industries plc v Dickman* [1990] 2 AC 605 does not provide a single tripartite test requiring consideration of proximity, foreseeability and whether it is fair, just and reasonable to impose a duty in every case. Rather, the courts must use existing authorities to apply established principles to the facts of the case. It is only in a novel type of case, where established principles do not provide an answer, that the courts need to ask whether a duty of care should be recognised, developing the law incrementally and by analogy with existing authority. The exercise of judgment in those circumstances involves consideration of what is fair, just and reasonable.
5. At trial, Ms Gumbel identified three potential routes to a duty of care:
   1. The claimant was a patient of the defendants (or at least the second defendant) so that the case falls within the scope of the established duty of care arising out of the doctor-patient relationship.
   2. The forensic psychiatry unit of the second defendant assumed responsibility for the welfare of the claimant, both in the context of providing family therapy and through her long-standing relationship with the team caring for XX and her involvement in his rehabilitation programme.
   3. If neither of the above routes are found to apply, by the application of established principles to the facts of this case by incremental extension (as explained by *Caparo v Dickman* and *Robinson v Chief Constable of West Yorkshire*) .

The defendants’ position

1. The defendants are jointly represented and have stood together in their defence of this claim. The claimant’s case was opened on the basis that the three defendants were to be treated as a multidisciplinary team with shared responsibility for the clinicians, therapists and social workers who made up that team.
2. It is established that the duty of care to a NHS patient is owed by the hospital trust; that such duty must be considered in the round and that it applies to medical and non-medical staff alike (see *Darnley* (supra) at [17 - 19]). The defendants do not invite me to seek to apportion between them if I find that liability is established. However, that does not avoid the need to look at where any duty lay and to consider the scope of any duties owed by the various teams and individuals involved.
3. For the defendants, Mr Havers QC argued that this is plainly a novel claim. The defendants deny that the claimant was their patient, rather she was a third party to the relationship between each of them and XX. Even if she was to be treated as in a relationship equivalent to that of a patient so far as family therapy was concerned, the scope of any duty arising from that could not encompass this case. The defendants also deny that there was any assumption of responsibility. Put simply, the defendants say that this is a novel case involving negligent omission in respect of which no duty has ever previously been recognised by the courts. Mr Havers contends that the court should not impose a duty of care in this case. Even if the claimant can establish the necessary proximity and foreseeability of harm (which is not conceded), it would not be fair, just and reasonable to impose a legal duty in the circumstances of this case. Mr Havers advances policy reasons for not imposing a duty to the claimant, placing particular reliance on the duty of confidence owed by the defendants to XX and the conflict between that duty and the duty the claimant contends for.

The court’s approach to the existence and scope of any duty of care

1. I must consider whether a duty was owed to the claimant and, if so, the scope of the duty within the factual matrix of this case. As Lord Woolf MR said in *Kent v Griffiths* [2001] QB 36 [37]:

“In these difficult cases it is necessary to examine the facts in detail. … Before you can apply one case by analogy to another you need to be clear as to the facts to which you are applying it.”

1. While it may be reasonable to consider the possible wider implications of recognising a duty not previously recognised by the courts, it would not be appropriate for me to attempt to define the limits of any duty of care owed by doctors to those who are not their patients. That is not the way in which the incremental development of the common law operates. I am required only to decide whether, on the facts of this case, a relevant duty was owed to the claimant. If so, I must resolve whether there was an actionable breach of that duty.

The duty of confidence and the professional guidance

1. It is very well-established, and not in dispute, that medical professionals generally owe a duty to maintain confidence in information about a patient’s health and treatment.
2. There is a strong public interest in respecting medical confidentiality which extends beyond the privacy of the individual patient. In *Z v Finland* (1998) 25 EHRR 371, the European Court of Human Rights stated [95]:

“Respecting confidentiality of health data is a vital principle in the legal systems of all the Contracting Parties to the Convention. It is crucial not only to respect the sense of privacy of a patient but also to preserve his or her confidence in the medical profession and in the health services in general. Without such protection, those in need of medical assistance may be deterred from revealing such information of a personal and intimate nature as may be necessary in order to receive appropriate treatment and, even, from seeking such assistance, thereby endangering their own health and, in the case of transmissible diseases, that of the community.”

1. It is equally well-established that the duty of confidence is not absolute. In certain circumstances, the public interest in disclosure may outweigh the public interest in maintaining confidentiality. (See, for example *W v Egdell* [1990] 1 Ch. 359, where the Court of Appeal held that the public interest in protecting others against possible violence outweighed the public interest in maintaining confidentiality so as to justify a psychiatrist instructed by a patient subject to a restricted hospital order disclosing his report to the patient’s responsible clinician although doing so was contrary to the patient’s interests.)
2. Recognising the competing public interests that may arise in relation to medical confidentiality, the General Medical Council have issued guidance for doctors. The GMC Guidelines have evolved over time, taking account of developments in medicine and in the law. They are likely to continue to do so. The most recent guidelines (“Confidentiality; good practice in handling patient information”) were issued in 2017. They note that the law governing the use and disclosure of personal information is complex but give practical advice on applying ethical and legal principles in practice. It is notable that the 2017 guidelines contain specific guidance about the disclosure of genetic and other shared information, which had not featured in earlier guidelines.
3. The timeframe with which this case is concerned means that it is necessary to look to two earlier versions of the relevant guidance. That published in 2004 (“Confidentiality: Protecting and Providing Information”) was in force until 11 October 2009. New guidelines (“Confidentiality”) came into effect on 12 October 2009. All the guidelines start by acknowledging that patients have a right to expect that information about them will be held in confidence by their doctors but recognise that there will be circumstances where there is a public interest in disclosing information even though the patient does not consent.
4. The 2004 guidelines stated [22]:

“Personal information may be disclosed in the public interest, without the patient’s consent, and in exceptional cases where patients have withheld consent, where the benefit to an individual or to society of the disclosure outweigh the public and the patient’s interest in keeping the information confidential. In all cases where you consider disclosing information without consent from the patient, you must weigh the possible harm (both to the patient, and the overall trust between doctors and patients) against the benefits which are likely to arise from the release of information.”

Further advice about disclosure where a patient has withheld consent was to be found at [24]:

“In cases where there is a serious risk to the patient or others, disclosures may be justified even where patients have been asked to agree to a disclosure, but have withheld consent (for further advice see paragraph 27).”

That advice read [27]:

“Disclosure of personal information without consent may be justified in the public interest where failure to do so may expose the patient or others to risk of death or serious harm. Where the patient or others are exposed to a risk so serious that it outweighs the patient’s privacy interest, you should seek consent to disclosure where practicable …. If you seek consent and the patient withholds it you should consider the reasons for this, if any are provided by the patient. If you remain of the view that disclosure is necessary to protect a third party from death or serious harm, you should disclose information promptly to an appropriate person or authority. Such situations arise, for example, where a disclosure may assist in the prevention, detection or prosecution of a serious crime, especially crimes against the person, such as abuse of children.”

1. The 2009 guidelines recognised the competing public interests, before stating [37]:

“Personal information may, therefore, be disclosed in the public interest, without patients’ consent, and in exceptional cases where patients have withheld consent, if the benefits to an individual or society outweigh both the public and the patient’s interest in keeping the information confidential. You must weigh the harms that are likely to arise from non-disclosure of information against the possible harm, both to the patient and to the overall trust between doctors and patients, arising from the release of that information.”

Further guidance about disclosures to protect others was set out at [53]:

“Disclosure of personal information about a patient without consent may be justified in the public interest if failure to disclose may expose others to a risk of death or serious harm. You should still seek the patient’s consent to disclosure if practicable and consider any reasons given for refusal.”

In the following paragraph, examples were given relating to the prevention, detection and prosecution of serious crime.

1. The disciplines with which this case is particularly concerned, genetics and psychiatry, are areas in which specific guidance has been given by relevant professional bodies. In the field of genetics, the Royal College of Physicians, the Royal College of Pathologists and the British Society for Human Genetics Guidance published a report of the Joint Committee on Medical Genetics entitled “Consent and confidentiality in genetic practice: Guidance of genetic testing and sharing genetic information” in 2006. That guidance stated [2.5.3]:

“The Human Genetics Commission, the Nuffield Council on Bioethics and the GMC have all expressed the view that the rule of confidentiality is not absolute. In special circumstances it may be justified to break confidence where the aversion of harm by the disclosure substantially outweighs the patient’s claim to confidentiality. Examples may include a person declining to inform relatives of a genetic risk of which they may be unaware, or to allow the release of information to allow specific genetic testing to be undertaken.

Before disclosure is made in such circumstances, an attempt should have been made to persuade the patient in question to consent to disclosure; the benefit to those at risk should be so considerable as to outweigh any distress which disclosure would cause the patient; and the information should be anonymized and restricted as far as possible to that which is strictly necessary for the communication of risk.

We recommend that before disclosure is made when consent has been withheld, the situation should be discussed with experienced professional colleagues and the reason for disclosure documented. Current GMC guidance states that the individual should generally be informed before disclosing the information.”

1. The Royal College of Psychiatrists Guidelines 2006 (“Good Psychiatric Practice: Confidentiality and information sharing”) provided guidance to psychiatrists faced with instances where they were required to make a judgment on whether or not the public interest served by disclosure outweighed the duty of confidence. The guidance made it clear that each case must be considered on its merits and continued:

“Decisions to disclose patient-identifiable information apart from the few statutory exceptions… are matters of judgement – judgement that may be finely balanced. Such balancing would need to take into account the various legal responsibilities at stake, including the duty of confidentiality to the patient and the public interest in the health service maintaining confidentiality. Consideration will need to be given to whether the harm that could result from disclosure (e.g. the possible harm to the relationship of trust or the likelihood of non-concordance with a programme of healthcare intervention in the future) is likely to be outweighed by the possible benefits. The potential benefits would need to be soundly grounded in the expectation that disclosure would have the desired effect (e.g. a significant reduction in the risk of harm).”

The guidance advises doctors to maintain documentary evidence of the balancing exercise undertaken and, if necessary, to seek legal or other specialist advice. It then continues:

“The doctor must be certain that the disclosure is in the public interest; if he or she cannot be certain of this then the patient’s confidentiality must be preserved.”

The evidence at trial

1. The claimant gave evidence on her own behalf. I found her to be an honest witness and accept that her account of events was truthful. I have the utmost sympathy for the claimant. She has experienced a dreadful series of events culminating in the discovery that she has the gene for Huntington’s disease. It was unsurprising that she displayed emotion and anger in the course of her evidence. While I am confident that I can rely upon the claimant’s factual evidence of past events, her evidence as to what she would have done had she been told of her father’s diagnosis earlier requires greater scrutiny. I say this not because I have doubts about the claimant’s credibility but because necessarily this evidence is affected by hindsight and the context of this claim. At times, I thought that the claimant was keen to have her say and make out her case. Her evidence displayed a degree of fixed thinking rather than a willingness to reflect upon the questions put to her. I make it plain this is not a criticism of the claimant, but it is something I have taken into consideration when assessing her evidence. With this in mind, I have looked at the evidence she gave from the witness box alongside more contemporaneous evidence contained in the medical records.
2. The defendants called the consultant geneticists who had been involved with XX’s case, Dr McEntagart and Professor Patton. I also heard from clinicians from Springfield, namely XX’s responsible clinician, Dr Olumoroti, consultant psychiatrist; Dr Beck, consultant clinical and forensic psychologist, who led the family therapy team; Ms Gill, consultant clinical psychologist, who provided individual therapy to XX and took part in family therapy sessions; and Dr Harvey, who was at the time a specialist trainee in forensic psychiatry and part of the family therapy team. The final factual witness was Ms Davies, who was employed by the third defendant at the time and was XX’s social worker.
3. Generally, I thought that the defendants’ witnesses did their best to give truthful and accurate evidence, acknowledging that their recollections were not perfect in view of the passage of time. There was a direct conflict in the evidence of the claimant and that of Dr Olumoroti about how XX’s diagnosis was disclosed to her. I prefer the evidence of the claimant on this issue, for reasons I shall explain below. That has caused me to look critically at Dr Olumoroti’s evidence on other issues and to test it against the contemporaneous notes. However, I have not found Dr Olumoroti’s credibility to be undermined generally. I thought that the defendants’ other witnesses, particularly Dr Beck and Dr Harvey, were willing to reflect carefully when answering questions. Both were frank in their evidence that they had not agreed with the decision not to disclose XX’s diagnosis to the claimant when she was pregnant, although they respected and abided by that decision.
4. I heard expert evidence from two consultant clinical geneticists instructed on behalf of the claimant, Professor Anneke Lucassen and Professor William Newman. The defendant relied upon expert evidence from Dr David Craufurd, a consultant in neuropsychiatric genetics. The claimant had permission to rely upon two experts in genetics given that the evidence of Dr McEntagart and Professor Patton also included some expert opinion.
5. I note that Dr Craufurd has particular expertise and experience in Huntington’s disease. Professor Lucassen and Professor Newman have less direct, current experience of patients with the condition. Professor Lucassen has a special interest in the ethical issues arising from genetic and genomic practice and was the inaugural chair of the ethics and policy committee of the British Society for Genetic Medicine, She is now the chair of that society and has published widely on the issue of familial communication of genetic information in academic journals. Professor Newman sees patients with a broad range of inherited disorders and had special responsibility for patients with inherited cardiac conditions. He has been significantly involved in the use of whole genome sequencing in clinical practice and has a special interest in genetic ethics.
6. When the claimant’s experts, Professor Lucassen and Professor Newman were giving evidence, I was struck by how difficult each of them appeared to find this case. Ms Gumbel acknowledged that this was so but invited me to allow for nerves. While both are leading experts in their field, they are not seasoned medico-legal experts experienced in giving evidence in court. I fully accept this and do make due allowance for nervousness. In fact, it is not uncommon for even the most highly regarded experts with national reputations to display nerves in the witness box. In my view, that is often the mark of an expert who will give carefully considered evidence and seek to provide the utmost assistance to the court. I would put the claimant’s experts into that category. However, I did feel that their obvious discomfort in answering some questions went beyond mere nerves and demonstrated how difficult the issues arising in this case are, even for experts who have a special interest in ethics in genetic medicine.
7. I also received expert evidence from two forensic psychiatrists, Dr Gwen Adshead, called by the claimant, and Dr Tim McInerny, for the defendants. Dr Adshead has significant clinical experience in forensic psychiatry. She also has a special interest in psychiatric ethics. She has published widely on the topic of confidentiality in mental health. She is registered as a group therapist and has experience of family therapy. Dr McInerny also has significant clinical forensic psychiatry experience. Unlike Dr Adshead, he is not a trained family therapist but has been involved in and has prescribed family therapy within his clinical practice.
8. In addition to the experts called at trial, I had written reports from Dr James Briscoe, consultant psychiatrist, who dealt with causation and condition and prognosis for the claimant. I also received written evidence from Mr Denbow and Mr Attilakos, consultants in fetal medicine and obstetrics. It was unnecessary to call any of those experts but where their evidence is of some relevance to the issues I have read and considered it.
9. I note that I received no expert evidence in relation to the role of Ms Davies or any other social worker. In reality, by the conclusion of the trial, it did not appear that the claimant was maintaining any specific allegations of negligence against the third defendant or any social worker.

The relationship between the claimant and the second defendant

1. The claimant’s pleaded case is that in 2009 she was attending Springfield as a patient for family therapy as well as attending multidisciplinary meetings in relation to her father’s care.
2. The relationship between the claimant and the second defendant was founded upon her being the daughter of their patient, XX. He was detained in the hospital pursuant to the sentence of the court. The claimant and her sister were acknowledged to be victims of XX’s crime. Their mother had been killed. As such, the Probation Service was responsible for providing victim contact reports to the second defendant through their Victim Liaison Service. Those reports were relevant to any conditions of discharge for XX. It was the claimant’s status as XX’s daughter and her role in his rehabilitation that brought her to Springfield, but she was more than simply a relative of the patient as she was also a victim of his crime. It was recognised that this created a complex situation in which the needs of the family had to be considered when planning for XX’s rehabilitation and potential discharge.
3. In October 2007, the claimant was invited to attend a meeting with XX’s responsible clinician (then Professor Eastman) and a social worker (Khalia Bennett). The purpose of that meeting was said to be:

“to collate a family history as well as giving you an opportunity to discuss any concerns or queries you may have.”

The claimant was also informed by way of a letter from Ms Bennett dated 1 November 2007 that:

“you are welcome to contact myself or any member of the multidisciplinary team if you have any queries or concerns you may wish to discuss, or if you feel there may be any way in which we could provide you with any additional support.”

1. During 2008, it was identified that family therapy might be helpful and towards the end of 2008, the claimant was invited to a meeting with the family therapy team. By letter dated 25 November 2008, Dr Harvey explained that the meeting was to discuss the process of family therapy and to explore whether it may be helpful for the claimant.
2. The claimant did not attend the first meeting. It transpired that XX had discouraged her from doing so. He explained to the clinicians that he wanted to protect his daughters and did not think they could cope with family therapy. Following the Claimant’s non-attendance at that first meeting, Dr Harvey wrote to her on 10 December 2008. Within that letter, he said:

“I understand that, as a family, you have all suffered a great tragedy and now face many changes and uncertainties in the future. We remain committed to offering you a time and space to come along and discuss your own feelings about these issues, in whichever forum you think will be most helpful.”

The claimant first attended a family therapy session on 30 March 2009. Her evidence was that she did not find family therapy particularly helpful. A session was arranged for 6 July 2009, but the claimant indicated that she would not be attending. The family therapy team used the time to discuss the situation. In a letter sent to the claimant after that meeting, Dr Harvey wrote:

“The free slot the family therapy team had this morning gave us the opportunity to think about how we might better meet your needs. We feel that we have not sufficiently understood, or focused on, the impact of your mother’s death upon you and your sister. … We would therefore like to offer a space for you, and [your sister] should she wish, to meet with the family therapy team without your father. The aim of this would be to focus on you and your sister’s needs more directly and could be kept confidential from your father.”

The next session took place on 8 October 2009 and there were further sessions on 5 November 2009, and 4 December 2009.

1. The family therapy team was led by Dr Beck and involved Dr Harvey and Ms Gill. Family therapy was offered as part of the therapeutic interventions provided to XX while he was a patient at Springfield. As such Dr Olumoroti received feedback from the team and monitored any impact on XX’s care. However, it would not be right to characterise Dr Olumoroti as supervising the family therapy.
2. There was clearly an overlap between XX’s clinical care and the family therapy. I note that Ms Gill was XX’s clinical psychologist and that Dr Beck described herself as being part of Professor Eastman’s team. I do not find it at all surprising that the boundaries between teams were blurred. Indeed, it seems to me that such an arrangement was entirely necessary in order to deliver a holistic approach to XX’s care and rehabilitation.
3. Somewhat surprisingly, Mr Havers suggested in his closing submissions not only that the claimant was not a patient for the purpose of family therapy but that neither was XX. That submission was inconsistent with the evidence called by the defendants. Dr Beck, Ms Gill and Dr Harvey all accepted that XX was a patient of the trust and that it was in that context that they saw him in family therapy.
4. The position is more complex in relation to the claimant. Dr Beck suggested that the question of whether the claimant was a patient for the purpose of family therapy might be a matter of semantics. Dr Harvey said in evidence that the use of the word “patient” was problematic, particularly within a forensic psychiatric service. I understand that. The claimant would not naturally be described as a patient of the mental health unit where her father was accommodated. No patient record was created for her whereas entries relating to the family therapy were made in XX’s medical records. However, in the context of family therapy, her role was not simply that of XX’s relative. The letters to the claimant from which I have quoted demonstrate that her participation was not solely for XX’s benefit but was also designed to focus on her own needs and to offer a therapeutic benefit to her.
5. At trial, the defendants’ expert, Dr McInerny acknowledged that the word “patient” could have different meanings in different contexts and accepted that “An individual who is in therapy is a patient.”
6. In their joint statement, the psychiatric experts, Dr Adshead and Dr McInerny agreed that the claimant was a patient for the purposes of family therapy. They also agreed that the responsible clinician would not take on the health care of family members but that relatives would need to be treated to a professional standard within the context of family therapy. Dr Adshead and Dr McInerny agreed that family therapists routinely weigh up the needs of different family members and “pay attention to the ordinary duties of care in relation to risk of harm to self or others”.
7. Although Dr Beck said in her statement that the claimant was not her “patient”, both she and Dr Harvey appeared to accept that they had a responsibility to the claimant in the way in which family therapy was conducted. Dr Beck, who was head of the family therapy team and whom I regarded as a very impressive witness, accepted a duty not to exacerbate the claimant’s psychiatric or psychological condition through the family therapy. She acknowledged that the team would seek to do no harm to the claimant. However, they would not take on the provision of more general psychological care for the claimant. If such was needed, they would signpost the claimant to other services. The claimant was in fact already undergoing her own psychological treatment elsewhere.
8. I find that the claimant was a patient of the family therapy team. Alternatively, even if the claimant was not strictly a patient of the second defendant, her relationship with the trust as a participant in family therapy was so directly analogous to that of a patient undergoing therapeutic intervention that an equivalent duty would arise in relation to the delivery of that therapy. Whether the claimant is described as a “patient” or a “participant” in relation to family therapy is, in my view, a matter of labelling and does not affect the substantive position as to the duty owed.
9. However, the fact that the claimant was participating in family therapy did not mean that she was in a doctor-patient relationship with Dr Olumoroti. He was not involved with the delivery of the family therapy. I am unable to accept the claimant’s submissions to the effect that, through participating in family therapy, the claimant became Dr Olumoroti’s patient. I accept Dr McInerny’s evidence, set out in his report, that:

“It would be clinically and ethically inappropriate to take on clinical responsibility and treatment of both a patient and their relative in forensic clinical practice.”

I find that Dr Olumoroti did not take on clinical responsibility for the claimant. The contact that occurred between them was in the context of the claimant being a relative of Dr Olumoroti’s patient, XX. The status of that relationship was not altered by the claimant’s participation in family therapy.

1. I shall return to consider the scope of the duty owed to the claimant in light of these findings.

The Claimant’s role in XX’s rehabilitation

1. The claimant impressed her father’s clinicians with her commitment to her father’s rehabilitation. They acknowledged that the situation was far from easy for her. She attended meetings at Springfield and she and her sister were an important part of the plan for XX’s discharge. Part of the purpose of family therapy was to support them to support XX. As early as February 2009, XX’s records reference the very early stages of potential discharge plans.
2. The defendants agree, based upon the expert evidence of Dr McInerny, that when the time came that XX was moving into the community, the diagnosis of Huntington’s disease would have had to be shared with the claimant. If XX had still not told her, his confidentiality would have had to have been breached at that stage by informing the claimant. It is accepted that she could not reasonably have supported XX in the community without knowledge of the diagnosis.

Events surrounding XX’s diagnosis and the defendants’ response

1. Following XX’s appointment at the neurological clinic at St George’s, Dr Marion wrote to Professor Eastman on 29 June 2009. She noted a family history, which included that XX’s mother had suffered falls since age 55 and died around the age of 59. Dr Marion recorded a clinical history consistent with Huntington’s disease and indicated that she would refer XX to Dr McEntagart. She suggested that if Huntington’s disease was confirmed, it might provide an explanation for his criminal act.
2. It appears that the correspondence from Dr Marion was not reviewed by XX’s clinical team at Springfield until 27 July 2009. A meeting between Dr Bethan Roberts, Dr Harvey and another member of the team took place on the ward that day. This was clearly a significant development with implications for XX’s care plan. It was noted that the Ministry of Justice would need to be informed of a confirmed diagnosis of Huntington’s disease. When XX was spoken to, he was hostile about the clinical diagnosis, suggesting that the observed symptoms were the result of excessive caffeine intake. He refused to go ahead with genetic testing.
3. XX discussed the possible diagnosis of HD with his psychologist Ms Gill in his weekly individual psychology session on 30 July 2009. The records note:

“His main worry was his daughters being possible carriers and how he did not want to have further negative impact on them after the devastating impact of the index offence. He was especially aware that the HD diagnosis could impact on their decision to have children or not. However, [XX] was willing to discuss the HD diagnosis with his brother who he felt was in a different position and who could then decide what action he would want to take.”

1. During a ward round on 5 August 2009, at which Dr Olumoroti and Dr Roberts were present, XX repeated that he did not want genetic testing. Dr Roberts noted that he had a clinical diagnosis of a choreiform disorder but that genetic testing was required to confirm the diagnosis. She noted:

“Implications for daughters given autosomal dominant nature of the condition.”

1. Dr Roberts reviewed XX on 20 August 2009. He did not want to go ahead with genetic counselling or testing. He said that he preferred to live with the uncertainty. He made it clear that he did not want to tell his daughters about the possibility he had Huntington’s disease. He said that it would be too much for them to deal with. He did not want to tell anyone else in the family as he did not want to upset them. Dr Roberts noted that he appeared to believe that if the diagnosis remained unconfirmed, he had no responsibility to tell anyone. Dr Roberts decided to write to Dr McEntagart for advice.
2. Dr Roberts’ letter of 20 August 2009 is agreed to be a careful and considered letter. It set out the history and noted that XX was refusing to undergo any genetic testing or to consider meeting anyone from the genetics team for counselling. It detailed the known family members and set out the daughters’ circumstances, including that they did not have any children. It continued:

“XX reports that he has told his brother about his diagnosis but has not told his daughters; this appears to be due to feelings of guilt that they will have to deal with another major life event in addition to dealing with their mother’s death and XX’s responsibility for this. We do not feel that we, as the psychiatric team, are in a position to be able to inform his daughters without breaching XX’s confidentiality. Obviously, if XX has a diagnosis of Huntington’s disease, this will have an enormous impact on his daughters in terms of their own risk of developing the disorder.

We would be very grateful for your opinion on how best to manage this difficult situation. Is there a duty of care to inform his daughters, and with which team does this duty lie? Should he change his mind about counselling and testing, how quickly might he be able to be seen?”

1. Dr McEntegart replied to Dr Olumoroti on 7 September 2009. In the meantime, following the MDT meeting on 2 September 2009, the second defendant’s clinical team had discovered that the claimant was pregnant. In a later report for the Mental Health Review Tribunal, Dr Olumoroti was to record that XX was stable throughout September. XX was concerned that his daughters should not be informed about the possibility of Huntington’s disease as he felt that they might “get upset, kill themselves or have an abortion.” XX’s continued refusal to tell his daughters coupled with the discovery of the claimant’s pregnancy led to Dr Roberts sending a letter by fax to Dr McEntagart on 18 September 2009, in which she sought urgent advice. It is not clear when the second defendant’s team received Dr McEntagart’s first reply (although it is date stamped 23 September 2009). Dr McEntagart responded to the second request for advice by telephoning Dr Olumoroti and following up with a letter dated 25 September 2009.
2. In her letter of 7 September 2009, Dr McEntagart noted that XX had been offered an urgent appointment which he declined. Responding to Dr Robert’s enquiry, Dr McEntagart wrote:

“To address the main issue of the letter, as Huntington’s disease is an untreatable condition, although of course it would be entirely appropriate for his daughter to be given this information, I do not believe that we have duty of care to override his confidentiality and disclose this information to them.”

She repeated the offer to see XX in the genetics clinic to discuss the pros and cons of genetic testing. She then continued:

“If he feels certain that he does not wish to have a genetic test done, a number of other options are available to him. Firstly, he could disclose the diagnostic concern to his daughters and they could come forward for a discussion in the Genetics Clinic. This is not ideal but we do find ourselves in this situation fairly often. The first difficulty is that without a molecular diagnosis in an affected member of the family interpretation of a negative result tells us that they will not develop HD but there remains a small risk that the familial condition is a different neurological disorder due to a different gene. In this situation the result would be falsely reassuring. The second issue is that if one of his daughters did opt for a predictive test for HD and was found to have an expansion in the gene then we will have diagnosed the condition by proxy in the father. In this scenario we would discuss how his daughters would deal with this if their father did not want to receive the information.

A further option that would be available to [XX] would be for him to give a blood sample that could be stored for his daughters’ use in the future and he could opt not to receive the results of the genetic testing himself.”

1. In her letter dated 25 September 2009, Dr McEntagart indicated that she had had further discussions with her colleagues and with Dr Marion in light of the claimant’s pregnancy. She had established that XX’s presentation was very typical for Huntington’s chorea. She then wrote:

“… we all felt that the approach we would take if he was our patient which currently he is not, was to try and work with [XX] to explain why it may be helpful for him as a minimum to at least disclose to his daughters the concerns that he may be affected by Huntington’s chorea and that this may explain what has gone on in recent years. However, if he absolutely refused to do this we would not breach his confidentiality and tell his daughters.

A number of other points for consideration arose regarding his insight, capacity to make such decisions and the current level of contact with his daughters. From our phone conversation it appears that he is trying to repair his relationship with his daughters and he feels this new development will hinder this. I understand that one of his daughters does attend the hospital and has been involved in some of the meetings to discuss her father’s progress. It seems that you feel he is capable of understanding and processing the information pertaining to his current situation. I also understand that you believe that he is talking to his brother and this may be helpful.”

1. Dr McEntagart then continued by suggesting that it may be possible to negotiate a way forward with XX and set out four possible options:

“1) He could meet with a member of our genetics team to discuss the pros and cons of genetic testing with the implications for himself and his daughters and take the DNA test to answer the diagnostic question with certainty and then share that information with them.

2) If his daughters would like him to be tested but he does not want to know the result he could give a blood sample for his daughters’ use and make a pact with them that they will not communicate the results to him.

3) He may decide not to give a sample but could still agree to disclose the concerns that he may be clinically affected by Huntington’s disease. His daughters could then request referral to the genetics service in their own right to discuss having a predictive test for HD themselves if this is what they wished. There are limitations to this sort of testing but this would be explained to his daughters. Again a pact of non-disclosure to their father might be appropriate.

4) If he is unhappy to consider any of the options above his daughters could still request referral to the genetics service if they raise concerns about the family history of medical problems in their father and paternal grandmother. From time to time we do meet families who come with a family history such as theirs and no possibility of obtaining full information due to family disharmony and we might discuss whether HD could be an explanation for the history. This situation is far from ideal but with appropriate counselling and explanation of the limitations of testing for HD in such a setting we might offer such a test.”

1. I found Dr McEntagart to be an impressive witness. She gave her evidence in a measured and considered way. No criticism is made of her on the claimant’s behalf. Dr McEntagart explained that, although XX had not become a patient of the genetic clinic, she was seeking to advise the psychiatric team as to how her team would proceed if he was their patient. It was not uncommon for the genetic service to be asked for such advice. Dr McEntagart said in evidence that views about when it might be appropriate to breach confidentiality have changed over time, “dramatically evolving” over the last ten years or so. However, she thought she would still reach the same conclusions today. Had XX been her patient, she would have tried to find a way to alert the daughters without a direct breach of confidentiality. She did not think that she would have breached XX’s confidentiality if they had not been able to negotiate a way around his refusal to consent. However, she had never come across a situation where it had not been possible to negotiate a solution.
2. Dr McEntagart explained that in discussing the case within her own team and with the movement disorder neurology team they were seeking to balance the benefits to the daughters through disclosure of the information against the benefit to XX in preserving his confidentiality. At the time, they felt there was no therapeutic benefit to the daughters because there was no known treatment or cure for the disease. She contrasted that to other conditions such as the BRCA1 gene linked to breast cancer, where knowledge could make a difference. Dr McEntagart recalled discussing the potential for the claimant to terminate her pregnancy but had not thought that would be termed a “therapeutic intervention” such as might justify breaching confidentiality. In her thoughtful and frank response to cross-examination, Dr McEntagart acknowledged that being deprived of the choice to have a termination could be harmful but said that she had not considered at the time that a mother with the gene might want to have a termination as a result of her own status, regardless of whether the foetus was affected. She had not viewed the case as one in which “serious harm”, within the meaning of the professional guidelines, might result from the non-disclosure of the information. It is to be remembered that she had not met the claimant or XX. She was not making the decision as to whether to disclose or not but was providing advice to Dr Olumoroti and his team.
3. Having received Dr McEntagart’s advice, Dr Olumoroti reviewed XX on 2 October 2009. XX provided an email from his brother dated 9 September 2009, which he relied on to dispute that he had Huntington’s disease. XX’s brother said that their mother had died at the age of 75 and that her movement difficulties were due to “physical wear and tear”. In light of the confirmed presence of Huntington’s disease in the family, I am unable to accept that the contents of that email were in fact accurate. It appears that it was written at the direction of XX and may have been a misguided attempt to please him. However, I do accept that an objective reading of the email at the time would cast some doubt on the clinical diagnosis as it suggested that XX’s mother had not had Huntington’s disease and the condition would be far less probable without a family history.
4. Dr Olumoroti showed XX a copy of Dr McEntagart’s letter. He went through the four options one by one. He had the sense that XX thought he was being “boxed in”. He became defensive and referred to an article in a newspaper suggesting it would be unlawful to breach his confidence. He made a veiled threat to sue. XX did agree to see Professor Patton from the genetics team (Dr McEntagart having gone on maternity leave). Dr Olumoroti assessed that this was the option that XX found most tolerable. XX also agreed to Dr Olumoroti meeting his brother to discuss how to proceed.
5. The medical records contain no explicit reference to Dr Olumoroti conducting a balancing exercise as to whether the suspected diagnosis of Huntington’s disease should be shared with the claimant. Dr Olumoroti did not refer to carrying out a balancing exercise in accordance with the professional guidelines in his witness statement. However, in evidence, he said that he had regard to the GMC guidelines and had discussions with his colleagues before taking the decision.
6. Dr Beck and Dr Harvey’s evidence was that they thought the claimant should have been told about the clinical diagnosis of Huntington’s disease. Dr Beck told me that she personally found the situation very uncomfortable. She said a lot of family therapy is conducted without full disclosure but said:

“…this has remained in my clinical memory as a difficult secret to hold”.

Dr Beck said that the point at which she strongly believed the claimant should be told was when she became aware that she was pregnant, to allow her the autonomy to make the decision about her pregnancy. However, she could not see any way to alert the claimant without explicitly breaching XX’s confidentiality. She was angry about not telling the claimant and voiced her anger. Despite her discomfort at withholding this information though, she still thought it right to continue with the family therapy.

1. Dr Harvey had also expressed the view that the claimant should be told, although his response was more measured. He said:

“I felt the balance was closer and perhaps should have fallen down on the side of disclosing”

Ms Gill would also have preferred that the claimant be told, but as XX’s psychologist she did not seek to persuade or encourage him to tell his daughters. She said that it was a complex, difficult situation and she regarded her role as laying out the options for XX rather than expressing a view one way or the other.

1. Dr Beck, Dr Harvey and Ms Gill were all clear that, although they were able to express their views and did so, ultimately the decision as to whether confidentiality could be breached had to lie with Dr Olumoroti as XX’s responsible clinician.
2. It is therefore clear that there were discussions of the competing viewpoints within the team and that it was recognised that deciding whether to tell the daughters was a difficult decision. This perceived difficulty led to the psychiatric team appropriately seeking advice from the genetics team. Dr Olumoroti sought to implement that advice by getting XX’s agreement to see Professor Patton.
3. Dr Olumoroti could not see any way that he could alert the claimant to the genetic risk without breaching XX’s confidentiality. He was concerned about the distress that might cause XX and had to consider XX’s physical and emotional safety. Mary Davies, XX’s social worker, confirmed that he had been suicidal when he was admitted to the ward and that he appeared very distressed and agitated about what he saw as undue pressure on him to disclose to his daughters.
4. Dr Olumoroti did not read Dr McEntagart’s advice as suggesting that he should implement option 4 by raising the matter with the claimant. Rather, he understood that if she raised concerns with him or another member of the team at Springfield that they could advise her to see her GP or to seek referral to a genetic clinic. He was not aware of her raising any relevant concerns. He said he had “a constant discussion with XX” about telling his daughters but XX was adamant they should not be told.
5. There is no evidence of the claimant raising any relevant concerns such as might have led to a discussion about genetic risks after Dr McEntagart’s advice was given. There is one potentially relevant entry in the records which pre-dates the advice, relating to a review on 7 May 2009 which the claimant attended. The note reads:

“Daughter reports that [XX] could easily lose his temper and at times find it difficult to control himself. She confirmed that grandmother had a similar gaits.”

1. Some time was spent during the trial in debating whether the entry should have read “grandmother had a similar gait” or “grandmother had similar traits”. However, I do not think this takes things further. This discussion took place about six weeks before XX saw Dr Marion and before any concern had arisen about managing the genetic risk. Professor Eastman was the responsible clinician at the time. I accept that Dr Olumoroti was not aware that the claimant had raised any concerns about her grandmother. It would be unreasonable to have expected him to go back through the notes looking to see whether she might have said anything before he took over XX’s care. A note made months earlier would not realistically have provided a way to initiate a conversation that might alert the claimant to the risk without breaching confidentiality.
2. Having made the referral to Professor Patton, Dr Olumoroti waited for that to take its course. He did not seek an expedited appointment or chase the testing. In effect, Dr Olumoroti handed over the management of XX’s suspected genetic condition to Professor Patton.
3. Professor Patton visited XX at Springfield on 27 October 2009. The family history which Professor Patton obtained from XX was consistent with the email from his brother and was not typical of Huntington’s disease. Clinical examination demonstrated ataxia and balance problems, but other typical symptoms of Huntington’s disease were absent. Professor Patton therefore thought that the suspected diagnosis was doubtful. That view is supported by the letter he sent once genetic testing had confirmed the diagnosis when he indicated that he had thought that such a diagnosis was less likely.
4. Professor Patton discussed with XX the implications of a confirmed diagnosis of Huntington’s disease for his daughters. He noted from what XX said that the claimant’s due date was in March and he believed her to be about 20 weeks pregnant. He raised the possibility of testing in pregnancy but suggested that it might already be too late for that. Professor Patton’s priority was to get XX tested. XX agreed to testing only on the basis that the results would not be shared with family members. That option was one of the standard options presented on the pro forma consent form used for seeking consent to genetic testing. Professor Patton’s impression was that if the test was positive XX would share that with his daughters “at some stage” but that he did not want the doctors to tell them.
5. Professor Patton’s said that he had conducted a balancing exercise about telling the claimant about the possibility of Huntington’s disease at that stage but that he considered it appropriate to proceed with testing XX on the basis that the results would not be disclosed. He would not have recommended telling the claimant while the clinical diagnosis was doubtful and when he believed her to be 20 weeks pregnant. He thought it would be very difficult to tell her at that time as there would probably not be sufficient time to get XX’s results back, test her and then test the baby in time to arrange a termination before 24 weeks. He accepted that it would have been theoretically possible to arrange urgent testing of the claimant but he had never come across a situation where someone had undergone a termination without testing the pregnancy and he felt it was realistically too late for that to happen. He pointed out that it was not just the time needed for the laboratory to do the testing. Time also had to be allowed for counselling before genetic testing. That could not be done in a matter of a few days.
6. Professor Patton said that a lot of patients will, like XX, initially not want to tell their family but that eventually it will come out. He had not met the claimant or her sister and did not really know what the family situation was. He knew XX was talking to his brother about the possible diagnosis. Professor Patton thought that getting XX tested was a major step forward in the process.
7. XX’s results were reported by the laboratory on 9 November 2009. No request for expedition had been made and they came to Professor Patton within the usual timescale. He arranged to give them to XX by visiting him at Springfield with a genetic counsellor on 10 December 2009. By then, it would have been too late for the claimant to undergo a termination. After XX was informed of his positive result, he saw Dr Olumoroti. He again stressed that he did not want his daughters to be informed. He said that he particularly did not want the claimant to know until after she had given birth. Dr Olumoroti made an entry in his notes to warn staff that it would be a breach of his confidentiality for family members or others to be informed without his consent.
8. It is apparent from the records that XX continued to maintain that his daughters should not be told of his Huntington’s diagnosis, despite ongoing discussions about that. In February 2010, he suggested that he would tell the claimant after she had given birth but in June 2010, he was still firmly asserting that he did not want his daughters to know. Details of the diagnosis and of XX’s refusal to share it with his daughters were included in the reports prepared for the Mental Health Review Tribunal. The claimant and her sister were not invited to attend the tribunal and did not see the reports.

The revelation of XX’s diagnosis to the claimant

1. Following the decision of the Mental Health Tribunal to direct XX’s discharge, Dr Olumoroti and a social worker (Khalia Bennett) attended the claimant’s home on 24 August 2010 to reassure the claimant. Her baby was then four months’ old. The claimant and Dr Olumoroti gave differing accounts of what happened during that meeting. Ms Bennett was not called to give evidence.
2. The claimant says that she had been angry that she had not been invited to the tribunal hearing. Dr Olumoroti dropped Huntington’s disease into the conversation then immediately said “Oh, your father didn’t want you to know this.” She said she was not upset when Dr Olumoroti revealed the Huntington’s diagnosis as she had no idea at the time that it was genetic. She replied, “Don’t worry, I will not say anything.” It was only after Dr Olumoroti and Ms Bennett had left that she looked Huntington’s disease up on the internet and was devastated to learn of its hereditary nature.
3. Dr Olumoroti’s evidence was that as he and Ms Bennett were leaving, the claimant asked him about the result of the Huntington’s test and he replied “positive”, immediately recognising that he should not have said that. He said that it was not him who had raised Huntington’s disease but the claimant. He did not know how she had discovered about her father’s diagnosis.
4. I find it much more likely that the claimant’s account of this event is accurate. In part, that is based upon my assessment of each witness’s oral evidence. The discovery that XX had a genetic condition was a very significant one for the claimant and is bound to stick in her mind. I had the impression that she was giving truthful evidence about it. Further, I have carefully reviewed the claimant’s medical records and find that they are entirely consistent with her account of first learning anything about Huntington’s disease on the date of Dr Olumoroti’s visit to her. The claimant was under the care of a specialist perinatal mental health team. In an entry dated 19 August 2009 in the contact notes of her perinatal mental health worker, Kathryn Siddle various concerns about her father and the outcome of the tribunal are noted but nothing which could conceivably relate to her having learnt that XX was being investigated for Huntington’s disease. On 24 August 2010 (the day after the meeting) Ms Siddle noted she was distressed because of an issue that “needs to remain confidential for the time being”. Subsequent entries confirm that the ‘confidential issue’ she was concerned about was the possibility that she had inherited the Huntington’s gene. The clear impression from the notes is that she knew nothing about Huntington’s disease until the meeting on 23 August 2010.
5. Her account that she was not upset in Dr Olumoroti’s presence because she did not then understand that Huntington’s disease was a genetic condition makes sense. I do note from the report of Dr Briscoe that the claimant told him that her father had said before Dr Olumoroti’s visit that there were “some other tests going on and I’ll talk to you about it another time.” It may very well be that she did ask Dr Olumoroti about these tests, but I am entirely satisfied on all the evidence I have seen and heard, that it was Dr Olumoroti rather than the claimant who first mentioned Huntington’s disease. The distress noted in her records from the following morning fits with her first learning of the genetic risk from her internet research after Dr Olumoroti’s accidental disclosure.
6. By contrast, I find Dr Olumoroti’s account surprising. He says that he accidentally let confirmation that her father had tested positive slip and then immediately left the claimant without making any assessment of whether she was upset. I find it very hard to believe that a responsible psychiatrist and social worker would act in that way, particularly knowing that the claimant had the care of a young baby. It is much more understandable that they would have remained with the claimant for a time and observed that she was not upset as was the claimant’s account.
7. Dr Olumoroti made an entry in XX’s notes on 24 August 2010 which is consistent with what he said at trial. It is clear that Dr Olumoroti was troubled by his accidental breach of confidentiality. He was worried about XX’s reaction. He may have reconstructed events to minimise his responsibility for the disclosure. He may well believe that the claimant mentioned the test for Huntington’s rather than just asking about “the tests” to which her father had alluded to. However, I prefer the claimant’s account that it was Dr Olumoroti who mentioned Huntington’s first.
8. In all the circumstances, I accept that the claimant learnt about her father’s diagnosis in the circumstances she described to me.

Events after the claimant learnt of XX’s diagnosis

1. I accept the claimant’s evidence that she was distraught after she had researched Huntington’s disease on the internet. She telephoned Ms Siddle for support at 8.15 a.m. the next day.
2. Dr Olumoroti saw XX to explain what had happened and thought that XX took the news well. He recorded that XX did not appear distressed and said he would not harm himself or commit suicide. Dr Olumoroti noted that XX thanked him for doing the difficult job of breaking the news for him.
3. On 25 August 2010, XX spoke to one of the genetic counsellors by telephone. They discussed the accidental disclosure to the claimant. The counsellor indicated that the claimant was welcome to call her, and the claimant spoke to her later the same day. The claimant was very upset. She indicated that she wanted to discuss the implications for herself and her sister before she told her sister. She said that her sister was ten-and-a-half weeks pregnant, but her father did not know of the pregnancy.
4. The claimant was referred to the genetic clinic by the counsellor, who wrote:

“[She] would like to come and discuss with you the implications of the possible diagnosis in her father for her and her sister in case there are any prenatal testing options her sister can avail of. She wishes to discuss this with you prior to informing her sister.”

The claimant spoke to the genetics counsellor again on 27 August 2010. The note of that conversation includes:

“Doesn’t feel she wants the test now and feels telling her sister now will not help her as she has always been more anxious than [the claimant].”

1. By letter dated 9 September 2010, the genetics service invited XX to reconsider disclosure to his family members. It was stressed that this was a choice for him and that whatever decision he made would be respected. Having received the letter from the genetics counsellor, XX continued to insist that his second daughter should not be told of his diagnosis.
2. The clinicians at Springfield continued to disagree about what should happen. Dr Beck raised her concerns within the hospital’s clinical and information governance structures. In an email dated 9 September 2010, she said that she had felt that it was in the claimant’s ‘vital interests’ to know about her father’s Huntington’s disease so that she could make a decision about whether to be tested and consider the implications for her unborn child. She said, “I thought she might choose to abort.” She then wrote:

“I spoke with More, the RMO, who said that the matter could wait until he returned from leave. I did not think it could given that [the claimant’s] ability and feelings about abortion would clearly change over the weeks he was away …. – weeks during pregnancy are of the essence in terms of decision to abort … I spoke with Crystal who took advice from Nigel Eastman and, I believe, Capsticks … Nigel was of the view that we should follow the principles adopted by the Genetics Clinic who consider it the realm of the patient with HC to decide who to inform. I disagreed with this, in writing, because as I said at the time, unlike ordinary cases of HC, [XX’s]’s HC may have contributed to his commission of a serious offence. I was over-ruled by all my colleagues in the Forensic Service (More, Crystal and Nigel) and [XX] was not informed.”

Professor Eastman disagreed with Dr Beck’s view. It was decided that the case should be discussed at a Clinical Ethics Committee meeting on 7 October 2010.

1. In advance of that meeting, the claimant had agreed with her father that her sister should not be told while she was pregnant. Those views were communicated to the ethics committee. The committee was chaired by Professor Eastman and made up of a range of independent members, including senior clinicians of different disciplines. Dr Beck, Dr Harvey, Professor Patton and Dr Olumoroti were in attendance. The committee decided by a majority vote not to disclose the information to the claimant’s sister.
2. The minutes of the meeting contain details of the discussion that took place about the history, nature of Huntington’s disease and the family dynamics. The family dynamics were thought to be unusual. It was recorded that:

“It is possible that the non-disclosure of the positive test result is either a benevolent act to protect the family or as an act of aggression.”

That accords with the picture that emerges from a review of XX’s records. His personality appears complex. There is no doubt that he could be difficult and controlling. However, at other times, he presented as pleasant and cooperative.

1. Ms Gumbel suggested that XX’s refusal to tell his daughters about his diagnosis was an act of control by a man with a history of domestic violence of the most severe kind. I accept that there are entries that could support such a view (including references to XX not wanting his daughters to undergo abortion). However, equally, there are entries which suggest that he did not wish them to be put through still more suffering after the death of their mother. I am unable to reach a clear conclusion as to what XX’s main motivation for not telling his daughters was. However, I consider that the conclusion in the minutes that it could either be a benevolent act or an act of aggression reasonably reflects the evidence contained in the notes. The conclusion of the committee was that it was not likely to be possible to understand the basis of the family dynamic.
2. Before the vote was taken the committee discussed the competing considerations, including:
   1. The harm and benefit in revealing the information to the claimant’s sister.
   2. The pregnancy being grounds for disclosure but that disclosure during pregnancy leads to distress and pressure to make a decision.
   3. The potential for the claimant’s sister to find out inadvertently; the fact that other family members already knew and the fact that XX said he would disclose when the time was right.
   4. The impact of secrecy on family relationships and the suggestion that the team were colluding in ‘secrets and lies’.
   5. The clash between confidentiality and the family therapy aspect.
   6. Whether Huntington’s disease played a part in the offence and whether that provided a reason for disclosure either on the basis of protecting the daughters from a risk of violence and/or reproductive choice.
   7. Some members of the team felt strongly that XX had a right to confidentiality whereas others thought that the rights of the daughters overrode the patient’s right to confidentiality.
   8. This was an unusual case but was it exceptional enough “for there to be deviation from the protocol and for confidentiality to be broken”?
3. The claimant has had a significant number of stressors in her life. In addition to the obvious trauma resulting from the killing of her mother and the issues in this case, she has experienced financial and employment problems. She is a single parent and there have been family court proceedings. It is unnecessary for me to say more about the claimant’s personal circumstances, particularly given the agreement on damages. I do note though that it is apparent that the claimant has had a very difficult time since the death of her mother.
4. The claimant attended the genetic clinic at Guy’s Hospital on 7 February 2011. At that stage, her sister still did not know about their father’s diagnosis. She requested a neurological examination before deciding about genetic testing. The claimant informed her sister about the family history fairly soon after her sister’s baby was born.
5. The claimant made a formal written complaint to the second defendant on 10 October 2011. Her sister was tested in December 2011 and found not to have the gene. The claimant reported at that time that “everyone wants her to be tested”. She was advised by the genetics nurse that if she was unsure, she should probably not proceed. In August 2012, she contacted the genetic service to book a test. The notes record that she had decided to take legal action and had been advised that she would need to undergo testing to make a sound case. The genetics nurse suggested this was not a good reason for testing. After further consideration, she confirmed in December 2012 that she wished to have the test, again referring to her solicitors’ advice that this was required for the claim. The nurse shared her concern about this, but the claimant said she needed to be tested as she felt that she was living her life as though she had Huntington’s disease anyway.
6. The claimant underwent genetic testing on 14 January 2013. The nurse noted at that appointment:

“Court case will proceed if she has a +ve test. Grounds for court case are that if she had known she had HD she would not have had [her child]. Hoping that if positive any compensation will help [her child] in the future.

When discussed further [the claimant] said that she wants test now irrespective of court case.”

1. In evidence, the claimant accepted that one of the reasons for having the test when she did was for this case, but she also wanted to plan for the future. She said she had not felt ready for the test earlier. It was not something she could “just do”. She did not know how she would cope with hearing that she was gene positive. She had been toing and froing for years. She was aware that the result could not be taken back once she had it. She said that there was not the same urgency once she had had her baby as there would have been antenatally.
2. The claimant received her positive test result on 28 January 2013.

The expert genetic evidence

1. The issues covered by the expert medical evidence were somewhat broader than typically seen in clinical negligence cases. In allowing the appeal against the striking out of the claim, the Court of Appeal indicated that this court was likely to be assisted by expert evidence in relation to the existence and scope of any duty of care, in particular in relation to some of the policy arguments raised by the defendants.
2. On the policy issues, I find a large measure of agreement between the geneticists, including Dr McEntagart and Professor Patton as well as the experts instructed on each side.
3. It is difficult to do justice to the considered views expressed by the experts within a short summary. However, I find the following key points emerge from the genetic evidence as a whole:
   1. Medical confidentiality is a profoundly important principle. Routinely overriding patients’ confidentiality would damage the relationship of trust between doctor and patient and may discourage people from coming forward to seek testing and treatment.
   2. A broad or indiscriminate duty to disclose genetic information to family members against a patient’s wishes would cause geneticists very great concern.
   3. Direct contact of at risk family members is not a routine part of genetic practice in the UK.
   4. Clinical geneticists do not consider that they have a professional obligation to warn all relatives of genetic risks. It would be wholly impractical and inappropriate to impose a duty on doctors to trace and inform all relatives about the potential risk to their health.
   5. Geneticists routinely consider the position of relatives and work with patients to encourage the communication of genetic information to family members.
   6. It is very rare for geneticists to encounter a situation where they need to consider a direct breach of a patient’s confidentiality. Even those with a special interest in ethics to whom the most difficult cases are referred, such as Professor Lucassen, will see such cases only very occasionally.
   7. Usually, a patient’s initial refusal to disclose genetic information can be worked around through negotiation. Even if the patient continues to withhold consent, it is often possible to alert relatives without a direct breach of confidence.
   8. The professional guidelines permit disclosure of genetic information if that would avoid a serious harm to another person.
   9. None of the professional guidance mandates a particular decision but the relevant guidelines require that clinicians should undertake a proper balancing exercise between the interests of the patient and the at-risk relative. (See the experts’ joint statement [2].)
   10. The decision whether to disclose to the claimant in this case was a difficult and complex one, requiring the exercise of professional judgment.
4. All the genetic experts agreed that if the claimant had been known to them, they would have tried to alert her to a possible familial risk. Professor Lucassen and Professor Newman suggested that this could have been done without a direct breach of XX’s confidentiality. However, when this was explored in cross-examination, they were unable to explain how that could have been done in practice. Professor Lucassen said this:

“I don’t know what would have been the ideal setup … I just envisage there would have been a possible way to do this. It is difficult for me to say quite how it would have happened in the forensic psychiatry setting and that is I think where this is… a really difficult situation because the clinical geneticists didn’t see XX, they only advised about what to do in this situation… I agree that would have been difficult. But I think it would have been possible.”

1. Professor Newman suggested in cross-examination that Dr Olumoroti could have invited a geneticist to attend Springfield to have a conversation with the claimant. He accepted he had not raised that in his report, nor could he think of an occasion where he had attended another hospital for a similar purpose. He said he had been thinking through different scenarios since writing his report and while listening to the evidence at trial. He said:

“I absolutely agree that this is extremely complex, challenging counselling. But it is what myself and my colleagues throughout the country train to do, to find ways to express information in a way that is understandable to patients about their inherited risks and about the conditions that may affect them, and then in particular circumstances, very particular circumstances, find ways of sharing information where there is the minimal amount of disclosure of information that others have expressed that they do not want disclosed.”

1. I am unable to find on the evidence before me that it is probable that the claimant could have been alerted to the genetic risk without a direct breach of XX’s confidentiality. The claimant’s evidence did not establish how this could have been done within the particular factual situation of this case. I accept the evidence of the defendants’ clinicians, including Dr Beck, that there was no way in practice to alert the claimant without breaching XX’s confidentiality. Had there been, I accept that the family therapy team would have done that.
2. I note that in their reports neither of the claimant’s experts criticised the actions of Dr McEntagart and Professor Patton. Rather, they suggested that the clinical team at Springfield were at fault in not properly implementing Dr McEntagart’s advice. At trial, there was some criticism of Professor Patton, centred around him not having seen the earlier correspondence before his visit to XX. The information he obtained from XX was that the claimant was 20 weeks pregnant. Therefore, when he saw XX he considered that realistically it was already too late for the claimant to be counselled and tested in time to undergo a termination. In fact, the claimant was only 18 weeks pregnant on 18 October 2009 and therefore the timescale was not quite as tight as he believed.
3. In her report, Professor Lucassen accepted that by the time Professor Patton first came into contact with the claimant, the claimant’s pregnancy was “too far advanced to make acute disclosure appropriate”. She sought to correct that in the witness box, explaining that she had now realised that there would still have been time for a termination to be considered. In re-examination, she suggested, for the first time, that Professor Patton ought to have considered option 4 and conducted a balancing exercise. Professor Newman also said in oral evidence that Professor Patton might have done more but his explanation as to why he had not said so in his report was unconvincing and I had the clear impression that he was not firm in his criticism of Professor Patton.
4. I have concerns about the way in which this evidence developed. Professor Patton gave evidence without knowing that he was subject to any direct criticism. The expert reports did not identify this, and it was not directly put to Professor Patton that he was at fault. In the circumstances, I am unable to give any significant weight to the allegation that Professor Patton was at fault. The two genetics experts instructed by the claimant had had ample time to consider his position after a full and careful review of the medical records. It seems to me that it is unrealistic to suggest that he should have recognised that there was a need to take urgent action at that stage. Professor Lucassen and Professor Newman had not recognised this when writing their reports after a full review of the medical records and no doubt very careful consideration of the facts. I do not believe that the criticism of Professor Patton, which developed late in the case, materially advances the claimant’s case and I reject it.
5. In providing their opinions on the actions of Dr Olumoroti and the team at Springfield, the expert geneticists have perhaps strayed into areas that fall outside their expertise. I note that the agenda for their joint statement encouraged them to do so. However, the conduct of the Springfield clinicians properly falls within the remit of the forensic psychiatric experts.

The psychiatric evidence

1. Again, there was much about which Dr Adshead and Dr McInerny agreed. The following undisputed points emerge from their evidence:
   1. The fact that a patient is detained under a court order does not alter the starting point that confidential information should not be disclosed without consent.
   2. The duty of confidence is not absolute and forensic psychiatrists may be required to disclose confidential information to effect safe clinical care and/or in the public interest.
   3. Forensic psychiatrists face particular ethical challenges in balancing the interests of their patients against the interests of third persons and the public interest.
   4. It is recognised in forensic psychiatry that professional duties are owed to multiple third parties, including victims, potential victims, relatives, the Ministry of Justice, Parole Board and other organisations within the criminal justice system.
   5. The professional guidance to psychiatrists does not mandate disclosure where there is a risk of harm to a third party but does require a balancing exercise to be performed.
   6. A forensic psychiatrist would not have been expected to be aware of the specific guidelines relating to disclosure of genetic information but would have been expected to seek and follow appropriate advice.
   7. This case presented an unusual situation for a forensic psychiatrist and involved a difficult and complex decision.
2. The psychiatric experts disagreed about whether Dr Olumoroti and the team at Springfield owed a duty of care to the claimant and, if so, whether that duty was breached. Dr McInerny did not think the defendants owed a legal duty to the claimant but in any event thought that Dr Olumoroti and his team had fulfilled their obligations, having regard to the relevant guidelines on confidentiality. Dr Adshead considered that the circumstances of this case mandated telling the claimant about Huntington’s disease. In effect, her evidence was that the balancing exercise could lead to only one outcome.

Duty of care

1. I turn then to the first of the essential issues that I must determine, namely whether the defendants owed any relevant duty of care to the claimant. If so, I must consider the nature and scope of the duty owed. This requires consideration of the three potential routes identified by the claimant.

Case 1: Duty owed as a patient

1. I have found on the evidence before me, and in accordance with the agreement of the psychiatric experts, that the claimant was a patient of the second defendant’s family therapy team. Even if better described as a “participant” in family therapy, I have found that to be directly analogous to the situation of a patient undergoing therapeutic intervention. The family therapy was provided by the hospital trust as part of the health service that it offered. Therefore, the duty owed by hospital trusts to patients to deliver their services competently applied to all those undertaking the family therapy. I regard that as applying established principles to a new factual situation (as in *Darnley*) rather than recognising a novel duty.
2. Although I have found that the second defendant did owe a duty to the claimant through her participation in family therapy, like Nicol J [22] I cannot see that this claim can properly be characterised as badly performed family therapy such as to bring it within the scope of the duty identified above.
3. The relevant information did not become known to the defendants within the context of family therapy. It was acquired in the context of the treatment and management of XX.
4. Participation in family therapy does not bring with it a right to receive confidential information about other participants. On the contrary, the evidence before me illustrates that the maintenance of confidences is often an important part of the family therapist’s role. The claimant was told that she could discuss matters in confidence without her father being told. It is obvious that this would also operate in reverse.
5. The family therapy team took the view that the claimant ought to have information about her father’s diagnosis. They did not tell her because the responsibility for deciding whether to maintain confidentiality in that information lay with Dr Olumoroti as the doctor responsible for XX’s clinical care. It is not part of the claimant’s case that they should have overridden Dr Olumoroti and breached confidence without his agreement. The reason the family therapists could not tell the claimant about XX’s diagnosis without the agreement of Dr Olumoroti was not that he was the clinician in charge of the family therapy but rather because he was XX’s treating clinician and had acquired the information in that role.
6. I have found that the claimant was not in a doctor-patient relationship with Dr Olumoroti. Further, I accept Mr Havers’ submission that, even as a patient of the second defendant, the claimant remained a third party to the relationship between each of the defendants and XX. (See *Powell v Boldaz* [1998] Lloyd’s Rep Med. 116, where the Court of Appeal confirmed that, when considering whether a duty of care was owed by a doctor to a relative of a patient, the fact that the relative happens also to be the doctor’s patient makes no difference to the position.)
7. Family therapy in a forensic setting is a specialised and complex intervention. It is very different from surgical or medical treatment aimed at providing a ‘cure’. It is readily apparent that the individual dynamics of a family will impact upon the aims and likely outcomes. Family therapy does not involve individual therapeutic work but rather focuses on the relationships between family members. As Dr Beck and Dr Harvey explained, family therapy explores whether relationships can be rebuilt to provide support in the future. However, the outcome is sometimes that relationships are not reparable. The work done is non-prescriptive. Dr Beck explained that the therapists will effectively be facilitating a conversation amongst the family rather than paternalistically intervening.
8. In her report, Dr Adshead explained that in this case, family therapy was offered to:

“(a) increase the chance of XX’s rehabilitation succeeding by (b) improving his relationships with his daughters and (c) giving his daughters space and time to talk about their experiences and feelings honestly, which (it was hoped) might help them to come to terms with their experience of what had happened. The therapy was explicitly intended to focus on trying to restore family relationships after XX had acted in ways that ruptured them; with an intention that restored relationships would aid his recovery and rehabilitation.”

1. I accept that it was hoped that the claimant would support XX when he was released into the community. The defendants’ expert, Dr McInerny, accepted that if the claimant was to support XX on his release she would inevitably need to know about his diagnosis at that stage. However, I am unable to accept that this fact materially altered the scope of the duty owed to the claimant in the context of family therapy.
2. It is notable that the claimant has advanced several different formulations of the scope of the duty alleged. To my mind, this demonstrates the difficulty in framing this claim as one of negligence arising in the context of the hospital-patient relationship. In her closing submissions, Ms Gumbel contended that the scope of the duty owed to the claimant as a patient was:

“(i) to provide competent support in the role of helping the claimant in coming to terms with the events that had occurred;

(ii) to help the claimant better understand why they had occurred; this included helping the claimant deal with her own feeling of guilt that she may have been instrumental in the killing by encouraging her mother to leave her father;

(iii) to help the claimant rebuild her relationship with her father;

(iv) to protect the claimant from suffering any further harm from her father including harm she might suffer from his controlling personality; and

(v) to help the claimant prepare to support her father in the community for which plans were being made from February 2009, sometime before XX moved to Ellis Ward.”

1. I consider this formulation of the scope of duty to be far too wide. It does not fit with Dr Adshead’s interpretation of the purpose of family therapy or with the evidence generally as to the role of family therapy. The duty owed to the claimant in the context of family therapy was quite simply to conduct the therapy with reasonable professional skill and care. Even if the wide duty contended for was accepted, it is doubtful that it could capture the claimant’s case that she should have been provided with information about her genetic risk to make decisions about her pregnancy.
2. In all the circumstances, I find that the breach that is alleged cannot fall within the scope of the duty owed to the claimant as a patient of the family therapy team (or as a participant in family therapy).

Case 2: Assumption of responsibility

1. An alternative analysis of my conclusion that the claimant was a patient for the purpose of family therapy would be that the second defendant assumed responsibility for exercising reasonable skill and care in undertaking family therapy so as to avoid harming her. That leads to the same position and supports my view that whether the claimant is labelled a “patient” or a “participant” (so far as the family therapy is concerned) really makes no difference.
2. The second limb of the claimant’s case goes wider than that. She contends that the defendants’ clinicians not only assumed responsibility for providing the family therapy but also assumed responsibility for deciding whether she should be told of her father’s diagnosis. Ms Gumbel contends that they did that by embarking on the investigation as to how to confront the “difficult issue” described in the correspondence and having discussions about whether the claimant should be told. In her written submissions, Ms Gumbel argued:

“The Claimant whilst not at the time aware her plight was being discussed must be assumed to have relied on such discussion about her being carried out responsibly and competently and in any event relied upon the Defendants not to allow her father to cause her further harm. The Second Defendant was both involved in protecting the Claimant from further harm caused by XX and had a special level of control over that source of danger.”

1. It seems to me that these matters are more properly considered under the third limb of the claimant’s case. Other than to the extent that I have found the claimant to have been a patient, or alternatively as a participant in family therapy, I do not consider that the claimant can rely upon an assumption of responsibility in the sense required to bring her within the established category of cases where a duty of care exists.
2. In the Court of Appeal, Irwin LJ said that he could not see a foundation for this part of the claim, and he would not have allowed the appeal against the strike-out on the basis of assumption of responsibility. He left open the possibility that this part of the claim might be justified on a closer analysis of the evidence. I do not consider that the evidence called at trial materially strengthens this aspect of the case, which I think can therefore be dealt with fairly shortly.
3. This is not a case falling within the principles of *Hedley Byrne & Co Ltd v Heller & Partners Ltd* [1964] AC 465 because there is no question of the claimant having relied on the defendants to undertake the balancing exercise as to whether she should be told of her father’s diagnosis. As is acknowledged, she knew nothing of it. I have already dealt with the scope of the second defendant’s assumption of responsibility in the course of family therapy. This did not extend to all aspects of the claimant’s well-being and could not extend, as is alleged, to preventing her father causing her any further harm.
4. The real thrust of the claimant’s case seems to me to be as follows:
   1. The defendants acquired genetic information about her father in the course of treating him.
   2. That information directly affected her.
   3. Not having that information could foreseeably lead to the claimant being unable to exercise her reproductive autonomy.
   4. The unusual factual matrix gave rise to a proximate relationship between the claimant and the second defendants.
   5. The defendants knew that the claimant had already suffered psychological harm and there was a foreseeable risk that withholding the genetic information would cause her further harm.
   6. The defendants actively considered the impact of the information on the claimant and whether it should be disclosed to her at a time when it was open to her to terminate her pregnancy.
   7. In the circumstances, the defendants owed her a duty of care in relation to the handling of the genetic information.
5. In my view, this does not involve the application of an established duty to a different factual situation (such as in *Darnley*) but is a novel claim in relation to which a duty of care has not previously been recognised. On that basis, the third limb of the claimant’s case must be considered.

Case 3: Should the court recognise a duty of care in this novel situation?

1. The claimant’s submissions under this third head identified that the court was being asked to consider the duty of a multidisciplinary team (including geneticists, forensic psychiatrists, family therapists and social workers) when confronted with the situation that a patient diagnosed with Huntington’s disease does not want his pregnant daughter to know of the diagnosis because, amongst other reasons, he does not want her to have a termination of pregnancy.
2. The duty which the claimant contends for under this limb was described by Ms Gumbel as:

“a duty to balance the Claimant’s interest in being informed of her risk of a genetic disorder against her father’s interest in having the confidentiality of that diagnosis preserved.”

Ms Gumbel limited the context of that duty to serious genetic conditions and first-degree relatives and suggested that it would be restricted to a duty to exercise care within the guidance given to doctors practising in genetics. Further, she acknowledged that the standard of care would be measured by reference to the well-known principles set out in *Bolam v Friern Hospital Management Committee* [1957] 1 WLR 582 and *Bolitho v City and Hackney Health Authority* [1998] AC 232.

1. On that basis, if the defendants properly considered and balanced the competing interests and decided not to disclose, they would have discharged the duty provided that the conclusion that the information not to disclose was reasonable. Here, it is the claimant’s case that, properly conducted, the balancing exercise permitted only one outcome, namely that disclosure should have been made.
2. Ms Gumbel did not identify any existing authorities in which a doctor or hospital trust had been found to owe a duty to a relative of a patient or to any third person outside the scope of the doctor-patient relationship. Mr Havers did highlight cases in which the courts have found that no duty was owed by doctors or health authorities to the parents of patients. (*Powell v Boldaz* (supra.) and *JD v East Berkshire Community Health NHS Trust* [2005] UKHL 23.) However, as Irwin LJ noted [48], the factual matrix in those cases was quite distinct from the present case. I note that the foreign authorities considered in the Court of Appeal were not cited in argument before me.
3. Mr Havers conceded that the existing authorities do not preclude the court finding that a duty is owed by a doctor to a third party. However, he stressed that, so far, no such duty has been found. It is necessary in this novel situation to look to the existing authorities for analogy. While Mr Havers accepts that it remains open to the court to find a duty by incremental extension, he contends that the duty contended for in this case would not be an incremental step. Rather it would have far-reaching implications and would represent a giant step inconsistent with the proper, incremental development of the common law. Applying the principles in *Caparo Industries PLC v Dickman,* Mr Havers argues that the court should not find that any duty is owed by a doctor or hospital trust to a relative of a patient or to any third party outside the doctor-patient relationship.
4. It seems to me that some of the arguments advanced on behalf of the defendants are addressed towards a duty far wider in scope than that contended for by the claimant. For example, the suggestion that it would be wholly unreasonable and impracticable to impose a duty on a doctor to conduct a search for all persons who might be affected by a genetic condition or infectious disease is unquestionably right (and agreed by all the experts). However, that is far removed from the duty which the claimant invites the court to recognise.
5. I must consider the question of whether a duty was owed to the claimant within the factual matrix of this case. While it may be reasonable to consider the possible wider implications of recognising a duty that has not previously been recognised by the courts, it would not be appropriate for me to attempt to define the limits of any duty of care owed by doctors to those who are not their patients. That is not the way in which the incremental development of the common law operates. I am required only to decide whether, on the facts of this case, a relevant duty was owed to the claimant.
6. The duty which is contended for is not a free-standing duty to disclose genetic information. Any duty to provide information and advice to the claimant in breach of XX’s confidence could only arise where the outcome of a proper balancing exercise required XX’s confidentiality to be overridden. In the Court of Appeal, Irwin LJ put it this way [23]:

“Of course, there is no simple duty of disclosure. But if the clinician conducts the requisite balancing exercise, and concludes that it falls in favour of disclosure then a professional obligation arises. The question is whether a breach of that obligation is actionable.”

1. Mr Havers does not accept that a professional obligation to disclose does in fact arise in those circumstances. He argues that the professional guidelines permit disclosure but do not mandate it. While it is right that none of the guidelines mandate a particular outcome, they do highlight that there are situations where disclosure contrary to a patient’s wishes may be justified. The guidance requires clinicians to balance the harms likely to arise from non-disclosure against the possible harm to the patient and the overall trust between doctors and patients from the release of the information. The outcome of the balancing exercise may be that the risk of harm in not disclosing is so serious that it outweighs the patient’s and the public interest in maintaining confidentiality. In those circumstances, the guidelines indicate that disclosure should take place. The Court of Appeal rejected the defendants’ argument that the guidance can operate only as a shield for the doctor and not a sword.
2. The question I am considering then is whether a duty of care was owed to the claimant in the particular circumstances of this case to properly balance her interest in being alerted to the genetic risk against the interest of XX and the public interest in confidentiality when deciding whether or not to disclose the information. I must decide whether recognising such a duty would represent a legitimate incremental step, or whether it would be a giant leap as the defendants suggest.
3. I acknowledge the defendants’ argument, supported by *Powell v Boldaz* and *JD v East Berkshire*, that the orthodox position is that proximity exists only as between a doctor and his patient and that the doctor’s duty of care does not extend to a third party. However, the Supreme Court in *James-Bowen* (supra, [29]) noted that in *JD* Lord Nicolls had referred to the fact that a doctor often owes duties to more than one person. That accords with the expert evidence in this case. The forensic psychiatrists agreed that professional duties were owed to multiple third parties.
4. The Supreme Court explained the decision in *JD* as being based on the seriousness of child abuse as a social problem demanding that health professionals acting in good faith in what they believe to be the best interests of a child should not be subject to potentially conflicting duties to parents when deciding whether a child may have been abused. It was said to be necessary to have regard to the competing underlying policy considerations when determining whether a duty of care may be imposed. The decision of the Court of Appeal in this case was specifically quoted as an example of a doctor owing duties to more than one person.
5. Wrongful birth claims involving failed vasectomies may be seen as further examples of doctors owing duties to more than one person (although there the interests of the female partner are aligned rather than in conflict with those of the male patient). See for example *Thake v Maurice* [1986] QB 644 and *McFarlane v Tayside* [2000] 2 AC 59. In both cases, the wife of a married man who had undergone a vasectomy was able to claim damages for a subsequent pregnancy. By contrast, in *Goodwill v British Pregnancy Advisory Service* [1996] 1 WLR 1397, there was no sufficiently proximal relationship between the defendants and a woman who had later entered into a relationship with a man who had undergone a vasectomy for a duty of care to be owed to her.
6. I conclude that the courts have been willing to recognise that a doctor or health authority may owe a duty of care to persons other than their primary patient but that such a duty is only capable of arising where there is a close proximal relationship between the claimant and defendant.
7. In the case of the first defendant (including their geneticists), I am unable to find that there was any close proximal relationship between them and the claimant. They knew of the claimant’s circumstances, having been consulted by the second defendant’s clinicians. That did not give rise to sufficient proximity for a duty of care to arise. The geneticists had not met the claimant. Until October 2009, they had not even had any direct contact with XX.
8. By the end of the trial, no specific allegations of negligence were made against the third defendant. Had the third defendant stood alone, I do not believe that the claim could have been maintained at all on the evidence before me. It seems to me that the inclusion of the third defendant in the proceedings does not advance the claimant’s case. Therefore, I think it is unnecessary to consider the third defendant’s relationship with the claimant further.
9. The position so far as the second defendant is concerned is different. As I have found, the claimant was a patient of the second defendant. Although I have found that the decision whether to provide her with information about her genetic risk lay outside the scope of the duty owed to her in the context of family therapy, her participation in the family therapy is an important part of the factual matrix. The second defendant’s clinicians had a significant amount of information about the claimant and her circumstances. They knew that she had suffered psychological harm as a result of her father’s offence and was in a vulnerable situation. They were working with her to help her understand and come to terms with the offence. They were also anticipating that she would support her father in the community and had previously shared medical information with her. They also knew that the claimant had very little support available to her. They knew of the family dynamics. Her mother had been killed and her sister was struggling with her own situation. They knew the claimant was not in a settled relationship. There was a direct line of communication with the claimant. Had they decided to disclose the information to her, the family therapy team offered a route to do so in a supportive environment. In those circumstances, there was a close proximal relationship between the claimant and the second defendant.
10. That the claimant was at risk of suffering harm if the information about her genetic risk was withheld from her was not only foreseeable, it was actually foreseen by the second defendant. That is clear both from the medical records and the evidence of the defendants’ witnesses at trial. Dr Olumoroti accepted in cross-examination that he had thought it might be harmful to the claimant if information was kept from her. He said he had discussed that with XX.
11. The criterion of reasonable foreseeability does not require that the precise harm or the precise mechanism of harm can be foreseen, only that some harm is foreseeable. Here though it is apparent from the evidence that the defendants had foreseen the possibility both of psychological harm and the loss of the opportunity to terminate the pregnancy (see Dr Beck’s email of 9 September 2010 in which she said she had considered at the time that the claimant might choose to abort).
12. Having found proximity and foreseeability on the facts, I turn to the question of whether it is fair, just and reasonable to impose a legal duty of care towards the claimant on the second defendant. It is important to address the issue of justice and reasonableness having regard to the duty that is actually contended for, rather than a wider hypothetical duty. I have already commented on the fact that some of the defendants’ arguments are not focused on the duty the claimant advances.
13. Before Nicol J and in the Court of Appeal, the defendants advanced nine policy reasons why a duty should not be imposed. Mr Havers’ closing submissions were refined to take account of the evidence and the way in which the claimant’s case had been put at trial. His reasons for maintaining that it was not fair, just and reasonable to impose the duty contended for are, in summary:
    1. The stark and direct conflict with the duty of confidence owed to XX, rendering doctors and healthcare professionals liable to be sued whatever decision they reached.
    2. The negative impact the imposition of such a duty would have on the duty of confidence and, in turn, the relationship of trust and confidence between doctors and patients.
    3. Third parties may not wish to receive the confidential information or may suffer harm as a result of receiving it. It would be difficult for a doctor to weigh that risk in the case of a non-patient.
    4. It is unclear how far the duty would extend, and it would potentially have enormous resource implications for the NHS.
    5. There is no need for the law to impose a legal duty where a professional duty already exists.
14. I accept, of course, that a duty to the claimant would conflict with the duty of confidence owed to XX. However, it has long been recognised that the duty of confidence is not absolute. This conflict does not preclude the recognition of a duty in this case (see *James-Bowen* [29]). It is not uncommon for doctors to face difficult decisions which could potentially leave them liable to be sued whichever way they decide. Further, I agree with Irwin LJ that incentivising obligations in one way but not the other may encourage rather than diminish defensive medicine. What is being proposed is a duty to balance risks and interests properly. When looking at the standard of care and any question of breach, I entirely agree with the observation of Irwin LJ (set out below) that the courts would allow considerable latitude to clinicians faced with the dilemma of conflicting obligations.
15. I do not accept that the imposition of a duty to the claimant would have such a negative impact on the duty of confidence as the defendants claim. Certainly, I do not consider that a duty to weigh up the claimant’s interests alongside the duty of confidence to XX would damage the relationship of trust and confidence between doctors and patients and/or be detrimental to the public interest. It is already established that the duty of confidence is not absolute and may be overridden on occasions. The professional guidance reinforces this. The proposed legal duty would simply recognise and enforce the need for the balancing exercise already identified in the professional guidance.
16. The defendants’ third argument about the risk that a third party may not want to receive the confidential information does not arise on the facts of this case. It might represent a hypothetical argument against imposing a broader duty to trace relatives of those with a genetic condition but that is not what this case is about. The second defendant had a proximate relationship with the claimant. Following *Montgomery v Lanarkshire Health Board* [2015] AC 1430, the common law firmly recognises personal autonomy in relation to medical risks. The old paternalistic view that a doctor might withhold information about risks because it is the doctor’s assessment that it is better for the patient not to know is no longer good law. Had the information about the genetic risk come to the defendants in the context of a patient relationship with the claimant, they would have been bound to disclose it. In fact, the defendants’ contemporaneous assessment was that the information should be communicated to the claimant for her sake. The difficulty was that the information came to the defendants in the course of treating XX and, absent his consent, its disclosure conflicted with his confidentiality.
17. The argument that it is unclear how far the duty might extend is, I believe, the “floodgates” concern identified in the Court of Appeal. Irwin LJ accepted that this would have to be considered very carefully, particularly given the principle that the common law of negligence should advance by incremental steps.
18. I do not accept that recognising a duty to this claimant on the particular facts of this case would open the floodgates to numerous claims. I have already identified that the factual matrix is unusual and that there was close proximity between the claimant and the second defendant. Recognising a duty to her is nowhere near the giant leap that might be required to recognise a duty to multiple relatives around the world. The failed vasectomy cases to which I have referred demonstrate that the potential flood can, and will, be held back by the operation of proximity. The fact that Mrs Thake could recover for her pregnancy following her husband’s failed sterilisation did not open the floodgates to claims by any women who might subsequently enter into relationships with men who had undergone vasectomies many years before.
19. I would readily accept that a duty that distracts medical professionals from the primary care of their patients or which places unreasonable demands on an already stretched National Health Service would be inappropriate. Finding the existence of a duty on the facts of this case would not have that effect. Professor Lucassen neatly addressed this point at paragraph 124 of her report when she said:

“Arguably, the time and resources spent in not informing ABC were in the instance much greater than had she been informed as part of the relationship she already had.”

I agree. The withholding of the information in fact caused a distraction and took up considerable time within XX’s clinical team. The imposition of a legal duty on these facts would not require a greater use of resources than the existing professional guidelines call for.

1. For my part, I would not address the floodgates concern by way of the tentative distinction suggested in the Court of Appeal of limiting the duty to genetic cases. I am inclined to agree with the defendants’ submission that, as Irwin LJ accepted might be the case, such a distinction does not stand up to close scrutiny. A close review of the guidelines and the expert evidence called at trial does not, in my view, support a distinction in principle between genetic medicine and other areas. The nature of genetics is such that information directly bearing upon a patient’s relatives may arise more frequently than in other areas of medicine. However, I do not see why genetic information should be treated differently from other information which reveals a serious risk to another person. Forensic psychiatry is a good example of an area where clinicians are used to considering such risks. Suppose a patient disclosed auditory hallucinations telling him to physically harm his relative to his psychiatrist. I suggest it would be surprising if information of that nature was less susceptible to being subject to a duty to consider breaching confidence than a genetic risk.
2. In my view, the need for close proximity before a doctor is found to owe a duty to any person outside the immediate doctor-patient relationship acts as sufficient restraint on uncontrolled extension of the duty of care owed by medical professionals.
3. I reject the defendants’ fifth argument that there is no need to impose a legal duty where a professional obligation already exists. I see greater force in the contrary argument that recognising a legal duty in circumstances already covered by professional guidance represents only a modest incremental step. In *Montgomery* [93], the Supreme Court addressed similar arguments recognising the need to impose legal obligations which were consistent with the professional guidance in relation to consent. Imposing a legal duty which is consistent with the professional guidelines acts to enforce the guidance and potentially allows an injured party to recover compensation where their interests have not been properly considered in line with that guidance.
4. Further, as also recognised in *Montgomery*, under the stimulus of the Human Rights Act 1998, the common law has increasingly taken account of the fundamental rights recognised by the European Convention on Human Rights. The right to respect for private life under Article 8 covers both the right to medical confidentiality (*Z v Finland*) and the right to have information about one’s own health, including information relevant to reproductive autonomy (*AK v Latvia* no.33011/08). This was therefore a situation that engaged the Article 8 rights of both XX and the claimant. Recognising a common law duty to both parties to conduct a proper balancing exercise in accordance with the professional guidance is consistent with the way in which the law has developed to take account of the Convention.
5. For these reasons, I conclude that it is fair, just and reasonable to impose on the second defendant a legal duty to the claimant to balance her interest in being informed of her genetic risk against her father’s interest in preserving confidentiality in relation to his diagnosis and the public interest in maintaining medical confidentiality generally.
6. The scope of the duty extends not only to conducting the necessary balancing exercise but also to acting in accordance with its outcome. It would be irrational to hold otherwise. If a doctor is under a duty to perform tests, there is no point in those tests being completed but no action being taken in response. The same is true of the balancing exercise.
7. I do not accept that the fact that doctors may reach different conclusions when conducting the balancing exercise means that there can be no legal duty to disclose, if that is the conclusion in fact reached. Mr Havers argues that there can be no principled basis for allowing a claimant to sue for breach of duty if Dr A concludes in favour of disclosure but then for some reason does not disclose but not if Dr B decides against disclosure. However, that situation does not seem very far removed from one in which two doctors competently conduct an examination. Dr A identifies a rare condition requiring treatment; Dr B does not. If Dr A failed to refer for treatment, he or she could be sued. Provided the examination was not negligent Dr B could not. I think this is another hypothetical argument that is unlikely to arise and which in truth does not assist in determining the issues in this case.
8. The standard of care is to be judged by reference to the relevant contemporaneous guidelines. Any claimed breach of duty would necessarily be tested by reference to the *Bolam* / *Bolitho* principles. As Irwin LJ said [35]:

“Common law liability would be measured against those standards, with the relevant professional practice and guidance very much to the fore. Indeed it seems to me evident, given the difficulty of such decisions, that the Courts would allow considerable latitude to clinicians faced with such a dilemma.”

1. The guidance I am considering takes a conservative approach and generally sets a high bar for the disclosure of confidential information without the patient’s consent. The 2004 and 2009 GMC Guidelines provided for disclosure if necessary to prevent death or “serious harm”. The specialist genetics guidance required the benefit of disclosure to those at risk to be “so considerable as to outweigh any distress disclosure would cause the patient.” The Guidelines of the Royal College of Psychiatrists allowed for disclosure in the public interest to prevent “serious harm” to a third party but required the doctor to be “certain” that disclosure was in the public interest before confidentiality was breached.
2. If a defendant has conducted a balancing exercise properly in accordance with the professional guidance and has reasonably concluded that disclosure should not be made, they will have discharged their duty. No liability can then arise even though others may have taken a different view, allowing considerable latitude to clinicians faced with a difficult decision where the competing rights of two individuals are concerned. The courts will also recognise that taking a decision of this nature in the course of day-to-day clinical practice is very different from taking a decision after several days of evidence and submissions in the Royal Courts of Justice.
3. If a defendant has not conducted a proper balancing exercise (applying the *Bolam* test), the court will have to go on to consider what the defendant would have done if the exercise had been properly performed. If, on a balance of probabilities, the defendant would have disclosed, a potentially actionable breach will be made out. If the defendant would not have disclosed, the court will have to consider whether such a decision would have been negligent, in the sense that no responsible body of medical opinion would have supported it (applying *Bolitho*.)
4. In recognising a legal duty in these terms, the law is not imposing a new obligation on doctors or hospital trusts. Rather, the legal duty recognises and runs parallel to the professional duty to undertake a proper balancing exercise which all the experts in this case agreed already exists.
5. The legal duty is likely to arise only in limited factual circumstances where there is close proximity between the at-risk person and the medical professionals. Even where such a duty does arise, it seems to me that the circumstances in which it will give rise to a cause of action will be rare, for the following reasons:
   1. The standard of care will be measured by reference to the professional guidelines. The guidelines do not mandate a particular outcome. Further, they take a conservative position. Non-disclosure is the default position and the bar for breaching confidentiality is relatively high.
   2. A decision supported by a responsible body of medical opinion will not be considered negligent even though others may not have reached the same decision.
   3. The courts will recognise the pressures of day-to-day clinical practice and will afford considerable latitude to clinicians taking difficult decisions in that context.

Breach of duty

1. Having found that the second defendant owed a duty to the claimant and having identified the nature and scope of the duty and the applicable standard of care, I turn to consider whether there was a breach of that duty. Ultimately the question is whether the proper exercise of the duty should have led to the claimant being alerted of the genetic risk while she was pregnant. The claimant’s case is that proper performance of the duty owed to her mandated that outcome.
2. Had I found that the duty extended to the first defendant, I would not have found fault on the part of their clinicians. It is not in dispute that the advice given by Dr McEntagart was appropriate. I have explained above why I reject the criticism of Professor Patton, which only emerged in the course of the trial. None of the written expert evidence supports an allegation of negligence against any of the first defendant’s clinicians.
3. Likewise, there was no expert or other evidence to support a claim of negligence against the third defendant, and it appeared that the claimant had effectively abandoned any case against the third defendant by the end of the trial. I do not say that the claimant was wrong to include all three trusts, and I do not think this added materially to the costs or to the length of trial. However, given the evidence and my finding as to where the duty lay, the alleged breach of duty falls to be considered against the second defendant alone.
4. Essentially, responsibility for the decision not to inform the claimant lay with Dr Olumoroti. His actions are to be judged against the standards of a reasonable forensic psychiatrist. I bear in mind that any reasonable clinician is expected to seek and follow advice on matters that lie outside his specialism. The forensic psychiatry team did seek the input of the genetics team and in deciding whether there was a breach I must also have regard to their advice.
5. In her closing submissions, Ms Gumbel alleged that the family therapists were in breach of duty in failing to halt the family therapy in circumstances where the information being withheld from the claimant was so significant that the therapy could not properly continue. That specific allegation was not pleaded but was supported by the evidence contained in Dr Adshead’s family therapy report.
6. Leaving aside any concern about this point not being pleaded, I do not accept that continuing with the family therapy while the information was being withheld from the claimant did amount to a breach of duty. Dr McInerny did not accept that continuing with family therapy in these circumstances was inappropriate. In the joint statement, he said:

“… it raises clinical issues for the therapist but it does not mean that therapy cannot take place. Sometimes family therapy works with very difficult issues and secrets, including confidential information relating to a patient offender and/or their family that is not known by all, but it would be hoped that the therapy could establish a setting where such issues could eventually be disclosed and discussed.”

Having heard from Dr Beck and Dr Harvey, I am entirely satisfied that they carefully considered the position and made an appropriate decision to proceed. Dr McInerny’s support for that view represents a competent body of medical opinion.

1. In any event, there is no evidential basis for finding a causal link between this alleged breach and the claimed loss and damage because the evidence did not establish that halting the family therapy would have led to earlier disclosure.
2. The remaining allegations of negligence crystallised into essentially two limbs:
   1. failing to properly follow and implement the advice of Dr McEntagart, to allow the claimant to discover she had the Huntington’s gene in time to terminate her pregnancy;
   2. failing to conduct a proper balancing exercise as to whether disclosure should have been made without XX’s consent, it being alleged that disclosure was the only reasonable outcome.
3. No real criticism is made of the steps taken before receipt of Dr McEntagart’s letter of 25 September 2009. The second defendant’s clinicians had recognised that a diagnosis of Huntington’s disease in XX had significant implications for his daughters, had encouraged disclosure and had sought appropriate advice. Upon discovering that the claimant was pregnant, they had appreciated that the issue had become urgent and sought further advice.
4. It might be said that Dr Olumoroti did not appear to act with great expedition after his discussion with Dr McEntagart on 24 September 2009 and having received her letter dated the next day. Certainly, other members of the team appreciated that time was of the essence. Dr Olumoroti saw XX on 2 October 2009, but it was not until 15 October that he referred XX to Professor Patton. Three weeks had passed which was significant in the context of the pregnancy.
5. Dr Olumoroti had secured the agreement of XX to be seen by Professor Patton with a view to genetic testing taking place. That meant that the second and third options identified in Dr McEntagart’s advice effectively fell away. They were options to be considered if XX did not want to be tested himself but might be willing for his daughters to know of the risk. The reverse was true. It could not be said that Dr McEntagart’s option one had been fully effected as it was clear that XX remained fundamentally opposed to information being shared with his daughters. That was unlikely to change if his test was positive.
6. The question then arises as to whether it was appropriate to simply await the outcome of the referral to Professor Patton or whether further steps should have been taken at that stage.
7. Given that Dr McEntagart’s options 2 and 3 did not arise, the only remaining option identified in her advice was option 4. That involved the referral of the daughters to the genetics service if they raised concerns about the family history. I have found that the claimant did not raise any relevant concerns after Dr McEntagart’s advice had been given. I have further found that the earlier note about the grandmother did not reasonably provide a basis upon which Dr Olumoroti or his team could have been expected to implement option 4. Dr McEntagart confirmed that the way in which she had intended option 4 was “if the daughters raise concerns … take that as a lead”. On the findings I have made, that did not arise.
8. Dr McEntagart did not intend her advice to be taken as a series of steps to be worked through in order. I do not consider that it would reasonably be read in that way. All the geneticists agreed that had XX been their patient they would have tried to find a way to communicate the risk to the claimant. What Dr McEntagart was seeking to do was to set out a range of options for how the clamant might be offered access to the genetic service depending on the outcome of negotiations with XX. If the patient could not be persuaded through negotiation to agree to disclosure, a “softer route” could be found to get the information to a relative.
9. Professor Lucassen’s experience matched that of Dr McEntagart. She firmly believed that it would have been possible to find a way to communicate the risk to the claimant without directly breaching XX’s confidentiality. She would have favoured having a conversation with the claimant in which the family history was discussed providing an opportunity to air concerns. However, she accepted that such an option had not been proposed to Dr Olumoroti.
10. It struck me that Dr McEntagart and Professor Lucassen were both experienced and sensitive doctors with considerable experience in genetics. Each had plainly developed their skills in managing difficult situations concerning disclosure of genetic information. Having heard from them, I am unsurprised that their experience is that they can generally manage the disclosure of information to relatives without direct confrontation. However, I do not view their experience as universal. Professor Patton did not identify a way to alert the claimant without breaching XX’s confidentiality. Dr Craufurd, a geneticist with huge experience in managing Huntington’s disease, confirmed that generally the information will eventually be shared within families but that it might take a year or two for that to happen. He could think of cases where disclosure had been withheld for a few years. He felt it was particularly difficult before a confirmed diagnosis was made in the patient. He thought any suggestion that the claimant could be alerted through a discussion initiated by the medics felt “awfully similar to actually breaching XX’s confidentiality”. Professor Newman had less direct experience of managing these difficult situations. He acknowledged that this would have been a very complex counselling situation and that it may have been difficult to communicate information to the claimant without an apparent breach of XX’s confidentiality.
11. It was not specifically put to Dr Olumoroti that he should have found a way to alert the claimant without breaching XX’s confidentiality. The evidence of the family therapy team, which I accept, was that they simply could not think of a way to do that in practice. Otherwise, I have no doubt they would have done so. I have already said that, on the evidence before me, I am unable to find that it was probable that the claimant could have been alerted to her genetic risk without a direct breach of XX’s confidentiality.
12. It is also notable that the diagnosis of XX’s genetic condition was made within a highly unusual factual matrix. XX was not at liberty when he was referred for investigations. It is clear that he was not at all keen to attend the genetic service and that he could be extremely difficult. I am not convinced that even the most experienced geneticists are likely to have come across a comparable situation.
13. In the circumstances, it seems to me that the second defendant cannot be criticised for not finding a way to alert the claimant without directly breaching XX’s confidentiality. The real issue is whether, applying the relevant guidance, confidentiality should have been breached in time to allow the claimant to make a decision about whether to continue with her pregnancy.
14. I think it is fair to say that Dr Olumoroti does not appear to have fully recognised that time was running out to give the claimant information that might inform any decisions about her pregnancy. As Dr Adshead said, although XX had been referred to Professor Patton, the question whether to breach his confidence was still as live as it had been before. The fact that XX had agreed to be seen by Professor Patton did not address the claimant’s position as XX had made it clear that he would not disclose the outcome of his test to her. Therefore, the need to balance the claimant’s interest in knowing of the genetic risk had to be addressed at the stage where it was still open to her to terminate her pregnancy.
15. The expert forensic psychiatrists agreed in their joint statement that there was inadequate or absent evidence of a proper balancing exercise within XX’s notes. The record-keeping is agreed to have been “unimpressive”. There was no formal meeting to discuss whether there were grounds to breach XX’s confidentiality. The context of XX being in a forensic psychiatry ward is relevant. Dr Adshead made the point that it is very common within that environment for team meetings to be convened very swiftly to discuss difficult issues. Dr McInerny thought it was unfortunate that there was no Clinical Ethics Committee conference to discuss what to do when the claimant was pregnant in contrast to what happened in relation to her sister. Dr McInerny accepted that if the extent of the balancing exercise that was conducted was what was reflected in the clinical notes, that could represent a lack of competent care.
16. The psychiatric guidance places emphasis on the need for good record-keeping. Given the importance of this issue, it is unfortunate that there was not a more structured balancing exercise with a proper record being kept. The Clinical Ethics Committee conference when the claimant’s sister was pregnant both provided the structure for competing consideration to be aired and ensured that a proper record was maintained.
17. Although not in his statement, at trial, Dr Olumoroti gave evidence that he had conducted a balancing exercise in accordance with the GMC Guidelines and that he had taken account of the claimant’s pregnancy. He said that he recognised that if the claimant was to be told about the risk, she needed to be told as soon as possible in view of her pregnancy. He had in mind that if XX had Huntington’s, there was a 50% chance that the claimant had inherited the gene and if she had it her baby had a 50% chance of inheriting it. He had considered that it could be harmful to the claimant not to be told about the possibility of Huntington’s while she was pregnant if it later turned out she had the disease. However, he thought that breaching XX’s confidence could cause real distress to him. He had to consider his patient’s safety and XX had talked of killing himself. Dr Olumoroti did not feel able to breach his confidentiality. He had though had “constant discussions” with XX about the impact on his daughters and the potential for his relationship with them to be further damaged by withholding this from them. There had been many meetings and discussions within the team.
18. Even if criticism can be made of the second defendant’s decision-making process and record-keeping, the evidence does not establish that this affected the outcome. Dr Olumoroti decided not to breach XX’s confidentiality having taken the advice of Dr McEntagart and having heard the competing arguments of the family therapy team, including Dr Beck who strongly advocated disclosure. The subsequent decision of the Clinical Ethics Committee in October 2010 not to disclose to the claimant’s sister provides strong evidence of the likely outcome had a formal meeting been arranged to discuss the claimant’s case. Although Ms Gumbel argues that the claimant’s sister was in a different position, I conclude having considered the minutes that the balance was likely to have been the same in the claimant’s case. If anything, the arguments in favour of disclosure were stronger in October 2010 as XX had a confirmed diagnosis of Huntington’s disease by then, the sister was noted to be the only family member who did not know and XX was closer to discharge into the community. I find that had a formal meeting been arranged to conduct a full balancing exercise the outcome would still have been that the claimant was not told.
19. It follows that any failings around the process of decision making and record-keeping cannot give rise to a causative breach of duty. The real issue is whether disclosure was mandated while it was open to the claimant to terminate her pregnancy. To put it another way, was the decision not to disclose one which no reasonable forensic psychiatrist could have reached, with the assistance of a multi-disciplinary team and the support of the genetics service?
20. All the experts agreed that the decision was a difficult and complex one. I conclude that it fell into the category of decisions recognised in the psychiatric guidelines as “matters of judgment that may be finely balanced”. The starting point from those guidelines is that confidentiality must be preserved unless the doctor is certain that disclosure is in the public interest.
21. While all the experts agree that the claimant’s interest in disclosure had to be balanced against the maintenance of XX’s confidentiality, there is no clear consensus as to what the outcome of the balancing exercise should have been.
22. The claimant’s experts, Professor Lucassen, Professor Newman and Dr Adshead maintain that the claimant should have been told. However, I have already noted how difficult Professor Lucassen and Professor Newman appeared to find the issues in the case despite their considerable experience of ethical issues in genetics. At one point in her evidence, Professor Lucassen accepted that the decision was one about which reasonable and responsible clinicians could have taken a different view. Although that was contrary to other parts of her evidence, I do not think she had been confused by the question when she answered as she did. Professor Lucassen also agreed that responsible and reasonable clinicians could take different views as to whether depriving the claimant of the opportunity to exercise her reproductive autonomy in these circumstances could amount to “serious harm”.
23. Professor Newman was firmer in his oral evidence that there was only one reasonable outcome of the balancing exercise. However, in his written report, he had observed in relation to the decision concerning the claimant’s sister that the decision was made in good faith and:

“Differences in opinion are common in medicine, especially in complex situations.”

I noted that when asked about balancing XX’s interests, he replied:

“in my assessment and my assessment of what the balancing exercise would have been at that time, that the interests of ABC outweighed the interests of XX. I am not diminishing for a moment the fact that XX did have interests, that there were requirements to think about him and consider his needs. But I truly believe that ABC’s outweigh those.”

The way in which he answered, stressing that this was his assessment seemed to me to be consistent with room for a difference of opinion.

1. Dr Adshead said that “given the risk of serious harm and the guidance available”, she did not think any responsible psychiatrist could take a different view. I thought Dr Adshead was a good witness and I fully accept that she would have reached the decision to disclose. However, she was somewhat trenchant in her views. She placed significant weight on the claimant’s right to reproductive autonomy. I would not dissent from that, but she may have given less weight to other factors. In particular, she may have underplayed the risk of harm to XX and she had not mentioned the public interest in maintaining confidentiality in her report.
2. In her careful and considered evidence, Dr McEntagart confirmed her advice to Dr Olumoroti was that her team would not directly breach XX’s confidentiality. She maintained that this would still be her decision on the facts of this case and even though practice in relation to the disclosure of genetic information has moved on over the past 10 years. Her reasoning was that Huntington’s disease is untreatable and therefore the sort of therapeutic benefit that might exist in cases of treatable conditions did not arise here. She acknowledged that being deprived of the opportunity to undergo a termination could be harmful but had not seen this as reaching the bar of “serious harm” to justify disclosure under the terms of the guidance. She accepted that she had not considered the prospect of the claimant wanting a termination without testing the foetus as that was not a situation she had come across before. She was reflective and willing to acknowledge different viewpoints but her conclusion remained the same.
3. Dr Craufurd, an expert with regular, recent experience of dealing with families with Huntington’s disease gave clear evidence that he too would not have breached XX’s confidentiality. He placed importance on the public interest in maintaining confidentiality. He highlighted concerns that patients are often already reluctant to seek medical help and thought that this could increase if it was known that a diagnosis would be passed on to relatives. He accepted in evidence that he had placed less weight on the claimant’s reproductive autonomy and had given more weight to the potential damage to XX.
4. Dr McInerny took the view that Dr Olumoroti and his team had acted reasonably in not disclosing the information. His evidence was that he would not have made the disclosure at the relevant time, although he considered that it would have been necessary for the claimant to know of her father’s condition at the time he was to move into the community since it was anticipated that she was to be part of his support network. The claimant places significant weight on this point, arguing that if it was inevitable that she would have to be informed of her father’s diagnosis within a short time then it was illogical to place much weight on his right to maintain confidentiality.
5. I see some weight in that argument. However, I am unable to go so far as to say that the opinions of the defendants’ experts are illogical. It is a factor that might have been given more weight. On the other hand, I believe that the claimant’s arguments underplay the risk of harm to XX and of damage to the therapeutic relationship with his clinical team. The evidence does not support the claimant’s argument that XX withheld his consent to disclosure purely as an act of control. Other concerns were expressed, including that he did not think his daughters could cope with this information at that time and a concern that the claimant might kill herself. Even acknowledging as I do the importance of a woman’s reproductive autonomy, it cannot be said that the balance of risks and harm for her lay firmly in favour of disclosure. Discovering the genetic risk during her pregnancy would not have been at all easy. She would have been forced to face very difficult decisions. A father’s desire that she should not know at that time was not necessarily an act of control rather than an act of concern, as the Clinical Ethics Committee acknowledged.
6. In short, this was a difficult decision which required the exercise of judgment. The relevant guidelines for psychiatrists made it clear that confidentiality should not be breached unless the doctor was certain that this was in the public interest. The GMC guidelines supported breaching confidentiality to avert a risk of death or serious harm. There was room for reasonable disagreement as to how the judgment should be exercised. That is demonstrated by the lack of consensus in the medical opinion before me. The claimant has not demonstrated that the views of the defendants’ experts are illogical. I therefore conclude that the decision not to disclose was supported by a responsible body of medical opinion and cannot be considered to have amounted to a breach of the duty I have identified.
7. Although I have placed little weight on it when considering the issue of breach of duty, the defendants are entitled to point to the claimant’s own decision not to inform her sister during her pregnancy as undermining her case that a decision to disclose was the only reasonable conclusion. I fully accept that she was not in the position of a professional making a carefully balanced decision. She had recently suffered the shock of learning of the risk to her, which exacerbated her psychological condition. She could not be expected to make a dispassionate, reasoned decision in the way the clinicians could. I also accept that her sister’s circumstances were not identical to hers. On the other hand, she was considering her sister’s best interests and had a better understanding than anyone of the family dynamics and the impact of her father’s offending. The decision to be taken did not require specialist medical expertise. It does seem to me that it would be unduly harsh to hold the second defendant liable in negligence for reaching the same decision as the claimant did in relation to her sister.

Causation

1. I have considered the issue of causation independently from my finding that there was no breach of duty. The issue to be determined is whether, on a balance of probabilities, the claimant would have terminated her pregnancy if the genetic risk had been disclosed to her.
2. It is agreed that the claimant could have undergone a termination up to 24 weeks of pregnancy but not beyond that time. The latest date on which she could have had a termination was 6 December 2009. This would have required her to be referred for a termination by the end of November. Termination after 22 weeks (23 November 2009) would have required feticide to avoid the risk of a live birth. The fetal medicine experts agree that termination involving feticide is often a highly distressing experience. Naturally, the later in pregnancy a termination occurs the more distressing it is likely to be. This was an unusual case (Mr Denbow, the claimant’s expert describes the sequence of events as “unique”). Usually, when termination is considered for Huntington’s disease, parents know of the risk in advance and can arrange testing of the foetus with termination taking place, if required, at around 15 to 16 weeks. Mr Denbow accepts that in this case the timing of XX’s clinical and subsequent genetic diagnosis would not have left time for the usual pathway of clinical care.
3. The claimant’s evidence was that, upon being informed of her risk of having the Huntington’s gene, she would have been tested “without hesitation”. She said that, upon testing positive, she would then have undergone a termination without first testing the foetus. She explained that she would have done that because she would not have felt it was fair to bring a child into the world knowing that she was going to become unwell, particularly having watched her father’s health decline and when he had killed her mother when in the early stages of the disease. She said she was utterly traumatised, and the future was terrifying.
4. Realistically, in my judgment, a decision to inform the claimant of the genetic risk in response to her pregnancy could not have been made before early October. At that stage, her father had a working diagnosis of Huntington’s disease but had not undergone testing. Professor Lucassen confirmed that prior to XX’s molecular diagnosis, the claimant would have been warned of the significant risk of getting a false negative result (where the claimant was found to be clear of Huntington’s but in fact XX had a different genetic condition for which she had not been tested).
5. It is clear from the evidence of all the geneticists, coupled with the written evidence of the fetal medicine experts, that the timescale for the necessary counselling and testing of the claimant in time for her to undergo a termination would have been very tight, even without allowing for fetal testing. All responsible geneticists would have advised the claimant not to rush into genetic testing. All the experts acknowledged that if the claimant had learnt of her risk of Huntington’s during pregnancy it would have presented an extremely challenging situation.
6. In light of all the expert evidence I received, I conclude that if the claimant had deferred a decision about whether she would undergo testing herself until after her father had been tested, there would not realistically have been time for her to be tested and arrange a termination. XX’s results were reported on 9 November 2009, when the claimant was already 20 weeks pregnant. I do not think it realistic to suggest that XX’s results should have been expedited. He is unlikely to have cooperated with an expedited approach. Further, Dr McEntagart’s evidence was that testing takes two weeks and Professor Patton explained that the laboratory will run samples in batches to obtain better control so that it is unlikely an individual test could be brought forward. Therefore, if the claimant were to undergo genetic testing in time to arrange termination of her pregnancy, she would have had to do so without first having a molecular diagnosis for her father.
7. If the claimant had taken that course, I think it improbable that she would have had her own test result before 9 November. Had she been informed of the risk in early October, she would have had to undergo counselling. Dr McEntagart acknowledged that in urgent situations “crisis counselling” can be arranged. She would have been cautioned about the risks associated with testing without a confirmed diagnosis in her father and would have been advised not to rush into testing. She would have been counselled about the timescale and become aware that if she were to have a termination it would be at a relatively late stage, certainly after 20 weeks gestation. I conclude that after allowing for testing and counselling, she would probably have reached 22 weeks gestation so that feticide would have been required.
8. It is readily apparent that this would have been a very difficult position for the claimant to find herself in. The experts I heard from acknowledged that it would have been a challenging and distressing situation.
9. I find that it would have been theoretically possible, albeit presenting difficulties, for the claimant to be counselled and to undergo testing in time for her to have a termination if she had been told of her genetic risk in early October. However, if a decision had been deferred until after XX’s testing, it would have been too late. It is against that background that I must consider what the claimant would probably have done.
10. I consider that some of the questions put to the claimant missed the point and may have been seen by her as offensive. It does not seem to me that it was particularly relevant that she had been looking forward to the birth of her child, oblivious to her genetic condition, or that she was overjoyed to have her child. Nor do I consider it particularly relevant to consider what “most women” would do, given her unusual and tragic circumstances.
11. I have said I found the claimant to be a truthful witness. I accept that she truly believes that she would have been tested promptly and undergone a termination. However, her evidence was given with hindsight, knowing as she does now that she has the Huntington’s gene. I do not think that her evidence fully grappled with how difficult the situation would have been for her at the time or recognised the advice she would have received not to rush into testing during pregnancy. When the claimant did eventually decide to be tested more than two years after learning of her father’s diagnosis, her decision was materially influenced by the need to know whether she had the Huntington’s gene for the purpose of this claim. Her medical records contain evidence that the pursuit of this litigation has taken on great importance to her. She is extremely angry with the defendants. While all of this is understandable, I did feel that it may have coloured her evidence and given rise to an unwillingness on her part to contemplate the possibility that she may not have immediately undergone testing had she learnt of the genetic risk during her pregnancy.
12. The circumstances in which the claimant learnt of her father’s diagnosis were undoubtedly extremely difficult for her. She was at home, alone with her four-month old baby. Dr Olumoroti plainly should not have disclosed the information to her in the way that he did. Having done so, he ought to have made some arrangements for her to receive genetic counselling or other support. However, she spoke to Ms Siddle the next day and from 25 August 2010 she had access to the genetic counsellors.
13. The claimant knew that her sister was pregnant. She decided that her sister should not be told while she was pregnant. Ms Gumbel suggests that this is not relevant evidence. However, I do consider it material to the issue of causation. It appears inconsistent for the claimant to say that she would have reacted to being told during her pregnancy by immediately requesting testing, yet for her not to insist her pregnant sister be immediately informed so that she could be tested during pregnancy. The claimant explains this apparent inconsistency on two grounds. First, the manner in which she was told left her devastated and “not in the right headspace” to make any sensible decision. Second, her sister’s personal circumstances were not the same as hers and therefore different considerations applied.
14. In relation to the first point, I have no doubt that even a carefully managed disclosure would have been extremely difficult for her during pregnancy. Professor Patton indicated how difficult it was for anyone to learn of the risk of Huntington’s disease during pregnancy. It would be particularly difficult without a confirmed diagnosis in XX. Dr Briscoe said in his report that being informed of her father’s condition during pregnancy could have triggered a serious deterioration in her mental state. Certainly, it cannot be suggested that the claimant would not have been distressed to learn the news during her pregnancy.
15. In relation to the second point, I think it would be inappropriate to set out a detailed analysis of the claimant’s sister’s personal circumstances here. That would necessarily involve revealing confidential material about her in circumstances where she is not a party and has had no opportunity to make representations. Despite the anonymity order, there will be those who know the identity of the claimant and her sister and I think it unfair to reveal highly personal material about a non-party. However, having reviewed the evidence, I do not accept that the claimed differences can explain a wholly different approach. I note a relevant entry in the claimant’s notes (medical records page 2654) dated 10 August 2010 (two weeks before the accidental disclosure) which throws some light on the position as it appeared at the time, rather than with the benefit of hindsight.
16. In my view, the claimant’s reaction on learning the news during her sister’s pregnancy provides a good indication of her likely reaction had she received the news during her pregnancy. A letter from the genetic counsellor to the genetics team at Guy’s hospital dated 26 August 2010 indicates that the claimant wished to discuss the options for prenatal testing for her sister before considering informing her. Ultimately, the claimant decided not to tell her sister during pregnancy. Her formal written complaint dated 20 October 2011 makes clear reference to being denied the opportunity of testing to exclude Huntington’s in her child. That complaint (written before the claimant underwent testing) is not entirely consistent with the case now presented.
17. In my judgment, the claimant’s firm belief that had she been told during pregnancy, she would immediately have proceeded to be tested to allow her to undergo a termination if she was positive and without the need for fetal testing has developed in the context of her undergoing testing and discovering her status in contemplation of this claim. That is not to be critical of her or suggest she is deliberately tailoring her evidence. The point is simply that the decisions she would have had to make during pregnancy would have been made in a very different context. The other evidence suggests that, as in the case of her sister, she would initially have considered the prospect of fetal testing. She was likely to be advised about the disadvantages of rushing into testing during pregnancy and the difficulties of arranging fetal testing and reaching an informed decision within a tight timescale. She would also have been counselled about the additional risks associated with undergoing testing prior to a molecular diagnosis in her father.
18. The claimant would undoubtedly have faced a difficult decision. Dr Craufurd’s evidence was that termination of pregnancy was “pretty uncommon” in cases involving Huntington’s disease. He said the majority will simply carry on with the pregnancy. He accepted that the claimant had good reason to have a terrible view of the disease because of what her father had done but she would have been counselled to understand that violence is not a typical manifestation of the disease and that there were reasons to be more optimistic than she might think.
19. When the claimant did learn of her father’s diagnosis, she found the decision to have the test very difficult. She required a significant amount of counselling and was advised that if she was not sure she should not have the test. She said (more than once) “you can’t just sit down one day and say she will have the test”.
20. It is impossible for anyone to be certain about what the claimant would have done if faced with information about her father’s condition in early October 2009. However, I am not required to be certain but rather to decide the issue on a balance of probabilities. I have no doubt that she would have been distressed and traumatised. There would have been a significant time pressure. The situation would have been very challenging for the clinicians as well as for her. Although I accept that it is possible that she would have sought testing herself, I think it more likely that she would not have done so. In effect, she would have made the same decision as she later made for her sister that testing during pregnancy would not be desirable.
21. I conclude that the claimant has not proved that she would have undergone a termination if notified of the risk during pregnancy. Accordingly, even if she had established a breach of duty, she would not have succeeded on the issue of causation.

The Human Rights Act claim

1. Having considered the evidence in detail, I believe that the preliminary view of Irwin LJ that the Convention does not add anything to the common law, nor does it provide a basis for action if the common law does not, remains true.
2. I have considered the claimant’s Article 8 rights in determining the existence and scope of the duty which I have found was owed to her. I have acknowledged that her right to information about her own health is to be balanced against XX’s right to confidentiality. I have found that the common law is aligned to the position under the Convention in recognising a duty to the claimant as well as that owed to XX.
3. In rejecting the claimant’s case that there was a breach, I have found that the decision not to disclose to the claimant was a judgment open to the defendants after conducting a proper balance between the competing interests of the claimant and XX. This takes account of the considerable latitude afforded to clinicians taking difficult decisions.
4. I do not consider that the position is altered by viewing this as a human rights claim. For the same reasons as I have found that there was no breach of duty, I find that, although the claimant’s Article 8 rights were plainly engaged, the interference with them was justified and proportionate.
5. In truth, Ms Gumbel did not press the human rights claim in her closing submissions. Further, she accepted that the agreement reached between the parties as to damages did not permit recovery in the event that the claimant did not establish that she would have undergone a termination.

Conclusion

1. For the reasons set out in this judgment, I have concluded that the second defendant owed the claimant a duty of care to balance her interest in being informed of her genetic risk against her father’s interest and the public interest in maintaining confidentiality. The scope of that duty extended to conducting a balancing exercise and to acting in accordance with its outcome.
2. This duty arose on the particular facts of this case, which involved a close proximal relationship between the claimant and the second defendant and the foresight that she might suffer harm if not informed.
3. The duty I have found is not a free-standing duty of disclosure nor is it a broad duty of care owed to all relatives in respect of genetic information. The legal duty recognises and runs parallel to an established professional duty and is to be exercised following the guidance of the GMC and other specialist medical bodies.
4. I have not found sufficient proximity between the first defendant and the claimant such as to justify the imposition of a duty of care. Further, no sufficient evidential basis arose to maintain a claim against the third defendant.
5. Although aspects of the process of decision-making and the record-keeping may be subject to criticism, I have not found any actionable breach of duty on the part of the second defendant. The decision not to disclose was supported by a responsible body of medical opinion and was a matter of judgment open to the second defendant after balancing the competing interests.
6. Having analysed all the available evidence, I have found that the claimant has not established, on a balance of probabilities that she would have been tested and undergone a termination had the risk been disclosed to her during her pregnancy.
7. In all the circumstances, the common law claim fails on breach of duty and causation. Further, the alternative human rights claim cannot be maintained on the findings I have made. It follows that, although I have the greatest of sympathy for the tragic circumstances in which the claimant finds herself, this claim must be dismissed.
8. I thank Counsel and both legal teams for their assistance. It was readily apparent that both sides had done much work in considering and addressing the complex issues that arose in this difficult case.
